# Integrated Phylogenetic, Phytochemical, and In Silico Evaluation of *Equisetum diffusum* D. Don. Ethanolic Extract for Bioactivity

**DOI:** 10.1002/cbdv.202502873

**Published:** 2026-10-26

**Authors:** Nasir Assad, Muhammad Naeem‐ul‐Hassan, Muhammad Fayyaz ur Rehman, Yasir Assad, Hansa Gul, Marzia Batool Laila, Muhammad Nauman Khan, Zahida Nasreen, Muhammad Adnan, Alevcan Kaplan, Sezai Ercişli

**Affiliations:** ^1^ Institute of Chemistry University of Sargodha Sargodha Pakistan; ^2^ International Center for Interdisciplinary Research in Sciences The University of Lahore Lahore Pakistan; ^3^ Department of Zoology Hazara University Mansehra Pakistan; ^4^ Department of Zoology University of Mianwali Mianwali Punjab Pakistan; ^5^ Department of Botany Islamia College Peshawar Peshawar Pakistan; ^6^ Department of Botany University of Chakwal, Chakwal, Punjab, Pakistan; ^7^ Department of Chemistry Bacha Khan University, Charsadda Khyber Pakhtunkhwa Pakistan; ^8^ Department of Pharmaceutical Botany, Faculty of Pharmacy Dicle University Diyarbakır Turkey; ^9^ Department of Horticulture Agricultural Faculty Ataturk University Erzurum Erzurum Turkiye

**Keywords:** antioxidant activity, *Equisetum diffusum*, GC‐MS, molecular docking, phytochemicals

## Abstract

This study investigated the antibacterial and antioxidant potential of *Equisetum diffusum* D. Don. using in vitro assays, molecular docking, GC‐MS, HPLC, phytochemical screening, ADME, toxicity, and phylogenetic analyses. Phytochemical screening confirmed the presence of alkaloids, flavonoids, phenols, saponins, and fatty acids in the extracts. GC‐MS and HPLC analyses revealed the presence of bioactive compounds, including 7,9‐Di‐tert‐butyl‐1‐oxaspiro(4,5)deca‐6,9‐diene‐2,8‐dione, oleic acid, and bis (2‐ethylhexyl) phthalate. The oxaspiro compound exhibited the strongest docking affinity with antimicrobial (PDB: 1R4U, −7.4 kcal/mol) and antioxidant (PDB: 1AJ6, −7.3 kcal/mol) targets. In vitro antibacterial assays confirmed the inhibition of Gram‐positive and Gram‐negative strains, whereas DPPH, FRAP, and TFC assays validated strong antioxidant activity. ADME analysis indicated favorable pharmacokinetics for most compounds, although bis (2‐ethylhexyl) phthalate showed toxicity risks. Phylogenetic analysis revealed conserved protein targets across Equisetum species. These findings highlight *E. diffusum* as a promising source of natural antioxidants and antimicrobial agents, supporting further in vivo and formulation studies for pharmaceutical development.

## Graphical abstract

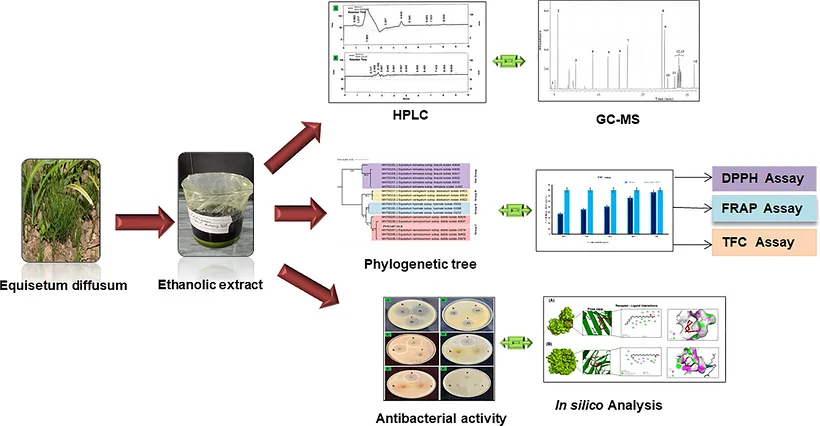

## 1 Introduction

For centuries, natural remedies have been used to enhance well‐being, and the effectiveness of contemporary medical research has heavily relied on the use of medications derived from natural sources. A significant number of antimicrobial substances have been identified in both artificial and natural sources to manage and regulate infectious pathogens [1]. Medicinal plants are important sources of pharmaceuticals used worldwide as alternative forms of medicine [2]. Approximately one‐quarter of medicinal drugs used today are derived from botanical sources [3].

The World Health Organization (WHO) reports phyto‐therapeutic substances from medicinal plants as treatment or prevention substances utilized by more than 80% of the global population [4]. Secondary bioactive metabolites, including flavonoids, alkaloids, terpenoids, phenolics, and glycosides, possess multiple beneficial properties, such as antioxidant, anti‐inflammatory, anticancer, anti‐aging, antiviral, antibacterial, antifungal, and antimalarial effects, which contribute to their extensive application [5]. These metabolites are extracted from plants using sensitive and sophisticated instruments. Approximately 20,000 plant varieties have been investigated for their therapeutic properties based on these specific traits [6]. The use of plant‐derived medicinal agents is supported by the increasing incidence of illnesses and advancements in the scientific understanding of herbal medicines as significant alternatives or adjunctive treatments for diseases [7]. The use of bioactive compounds obtained from plants or their synthetic counterparts in medicine has been enhanced by advances in phytochemistry and pharmaceutical chemistry. This is because medicinal plants possess a wider array and higher originality of compounds than any other source [8]. The initial step in the process involves extracting various phytochemical constituents, including phenolics, flavonoids, tannins, alkaloids, terpenoids, steroids, and glycosides, from different plant parts, such as leaves, stems, fruits, seeds, and roots. This extraction process eliminates unwanted substances and isolates bioactive natural products, which are then utilized as sources for the production of medicinal components [9]. Furthermore, the use of phytochemicals is now recognized as a potential holistic alternative method with few adverse reactions [10].

Phenolic phytochemicals derived from plants have a significant impact as antibacterial agents [11, 12]. Antimicrobial drugs degrade the protein constituents of the cell wall, interfering with enzyme activity and DNA and RNA replication [13]. Essential oils and bioactive phytochemicals have demonstrated substantial antibacterial activity against several strains of Salmonella and *Escherichia coli* [14]. Phytochemicals, such as polyphenols, phenolic acids, flavonoids, tannins, and anthocyanins, scavenge free radicals and exhibit antioxidant properties. Plants are often more affordable, readily accessible in local areas, and easily used as basic medicinal combinations for treating various illnesses than synthetic drugs. Consequently, plants may be analyzed to comprehend their therapeutic characteristics, as well as their safety, and effectiveness [15].

Standard approaches for evaluating compound bioactivity are expensive, time‐consuming, and labor‐intensive. The innovative technique of Computer‐Aided Drug Design (CADD) has proven itself as a potent replacement that boosts early drug discovery processes at reduced expense levels. The primary element of CADD performs in silico molecular docking operations to predict ligand interaction strength and protein‐binding conformations used for structure‐based drug design applications [16]. This methodology helps detect potential lead compounds and provides thorough information about protein–ligand bonds, the routes that drive these interactions, and compound–activity relationship (SAR) patterns. In silico assessments of the physical properties of substances combined with ADME characteristics support drug selection and promote experimental analysis during in vitro and in vivo evaluations of potential candidates [16, 17]. Therefore, these methods may assist in investigating the therapeutic capabilities of traditional medicinal plants, accelerating drug development, reducing costs, and enhancing safety [18].

*E. diffusum*  is the only existing member of the Equisetaceae family and the *Equisetum* genus, often known as horsetail, as shown in Figure 1S (Supporting File 1). It is local to the Himalayas. The plant is often referred to as the Himalayan horsetail and has been documented in ethnobotanical studies for its medicinal properties in treating various ailments [19]. Despite earlier reports on the antibacterial potential of *E. diffusum* extracts, the species remains insufficiently characterized through an integrated framework linking phytochemical composition with experimentally observed and computationally predicted bioactivity. The current study provides a comprehensive evaluation of the ethanolic extract of *E. diffusum* through phylogenetic relationships, phytochemical composition, and probable molecular interactions. We performed a phylogenetic study to ascertain the evolutionary connections of *E. diffusum* and other closely related species. We conducted phytochemical analysis using HPLC and GC‐MS techniques to identify and measure the chemical constituents in the ethanolic extract of *E. diffusum*. Furthermore, a computational blind docking method was used to assess the affinity of the compounds for protein binding. Later on, the in silico method was validated by conducting in vitro tests to evaluate the antibacterial effectiveness of these phytochemicals against bacterial strains of *Staphylococcus epidermidis* (ATCC 12228), *Listeria monocytogenes* (ATCC 13932), *Pseudomonas aeruginosa* (ATCC 10145), and *E. coli* (ATCC 10536). All studied phytochemicals were good, but the main activity was performed by Additionally, the in vitro antioxidant capacity was investigated using DPPH, FRAP, and TFC assays.

**FIGURE 1:**
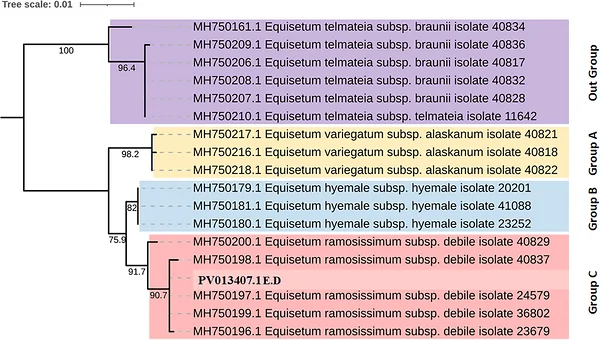
A phylogenetic tree indicates the evolutionary connection between *E. diffusum* (PV013407.1 E.D) and other species of Equisetum.

## 2 Results and Discussion

### 2.1 Phylogenetic Analysis

The phylogenetic analysis presented in the tree provides a detailed evolutionary relationship among different Equisetum species and subspecies based on genomic sequences. The tree is structured into three main clades (Groups A, B, and C) with *Equisetum telmateia* subsp. braunii as the outgroup, serving as an evolutionary reference point, as shown in Figure 1. The outgroup (purple) consists of *E. telmateia* subsp. braunii, which is genetically distinct from the other groups. Group A (yellow) consists of *Equisetum variegatum* subsp. *alaskanum* (A.A.Eaton ex Harriman) Hultén that forms a well‐supported (bootstrap 98.2) clade showing a close evolutionary associated genealogy. Group B (blue) is composed of *Equisetum hyemale* subsp. hyemale L., which has bootstrap support value of 82, a fairly well‐supported but slightly more variable group. Group C (red), consisting of *Equisetum ramosissimum* subsp. debile (Hance) Christenh., is strongly supported (91.7, 90.7 bootstrap values) and includes the sequence (PV013407.1. E.D), suggesting a close genetic relationship with this species. More recent molecular phylogenetic studies have shed more light on the phylogenetic relationships within the genus Equisetum. Recent molecular phylogenetic investigations also show that Equisetum is a monophyletic genus consisting of two main subgenera, Equisetum and Hippochaete. A study on the chloroplast genome *of Equisetum xylochaetum* Desf. found that Equisetum species share a conserved quadripartite structure of the chloroplast genome and include identical gene content and order [20]. Phylogenetic inference based on plastome protein‐coding regions supported the monophyly of the genus and suggested that *E. xylochaetum* is closer to *E. hyemale* than to *Equisetum arvense* L. These results support previous studies that proposed two main subgenus within Equisetum: Equisetum and Hippochaete and *Equisetum bogotense* Kunth as a sister to all other species and hence distinct evolutionary lineage [21]. Furthermore, systematic chemical analyses of different Equisetum species revealed different patterns of polar secondary metabolites, that is, hydroxycinnamic acids, flavonoids, that could act as chemotaxonomic markers as well as offer more clues in resolving the evolutionary divergence of these species. In addition, chemical studies have established unique secondary metabolite samples, including hydroxycinnamic acids and flavonoids, that are as chemotaxonomic markers of species at the molecular level [22]. Together these molecular and chemical studies contribute to a better comprehension of the phylogenetic and the evolutionary past of the Equisetum genus.

### 2.2 Phytochemical Screening

Phytochemical analysis of various *E. diffusum* extracts showed a wide range of solubilities. Alkaloids were commonly found in all extracts, demonstrating their solubility over a wide range, as shown in Table 1. Flavonoids were found in the aqueous and ethanolic extracts, both of which are highly polar, but were absent in acetone, n‐hexane, and chloroform extracts. Glycosides were selectively found in the n‐hexane extract, which matches their preference for nonpolar solvents. Lignin was detected in ethanolic, acetone, and chloroform extracts but was not found in aqueous or n‐hexane extracts. Saponin was found in the water and ethanol extracts but was absent in all the other solvents, indicating polar solubility. Coumarin was found only in the n‐hexane extract, which is consistent with its nonpolar characteristics. Phenols were found to be present in ethanolic, acetone, and chloroform extracts, suggesting moderate polarity. The majority of the extracts contained sterols, except for n‐hexane, which demonstrated broad solubility in various solvents. Leucoanthocyanin was exclusive to the chloroform extract, whereas fatty acids were found in the aqueous and ethanolic extracts, indicating their solubility in polar surroundings. Phytochemicals present in *E. diffusum*, such as alkaloids, flavonoids, and phenols, are known for their therapeutic properties, including anti‐inflammatory, antioxidant, and antimicrobial effects [23]. Alkaloids are often used for their analgesic and therapeutic properties, whereas flavonoids are valued for their antioxidant benefits, contributing to cardiovascular health and cancer prevention [24]. Phenols play a crucial role in preventing oxidative stress and are widely used in skin care products [25]. The presence of these compounds in *E. diffusum* highlights the plant's potential for use in traditional medicine, pharmaceutical applications, and as a source of natural health supplements.

**TABLE 1:** Phytochemical screening of *E. diffusum* extracts using different solvents, indicating the presence (+) or absence (‐) of various bioactive compounds. The analysis reveals distinct solvent‐dependent extraction of phytochemicals.

Sr. No	Phytochemicals	Aqueous extract	Ethanolic extract	Acetone extract	n‐hexane extract	Chloroform extract
1	Alkaloids	**+**	**+**	**+**	**+**	**+**
2	Flavonoids	**+**	**+**	—	—	—
3	Glycosides	—	—	—	**+**	—
4	Lignin	—	**+**	**+**	—	**+**
5	Saponin	**+**	**+**	—	—	—
6	Coumarin	—	—	—	**+**	—
7	Phenols	**+**	**+**	**+**	—	**+**
8	Sterols	**+**	**+**	**+**	—	**+**
9	Leucoanthocyanin	—	—	—	—	**+**
10	Fatty acid	**+**	**+**	—	—	—

### 2.3 HPLC Analysis

Based on the retention times and information regarding the compounds in Figure 2A,B, there is a noticeable overlap and differentiation in the chromatographic profiles, which likely reflects the complex nature of the compounds being analyzed. In Figure A (254 nm), compounds such as small phenolic acids, flavonoid derivatives, catechin derivatives, and tannins were detected, which are typical compounds that absorb at lower UV wavelengths. These compounds represent a wide range of polyphenols and related substances. Specifically, the compound detected at 3.967 min in Figure A is likely quercetin or kaempferol, both of which are flavonoids. In contrast, Figure B (326 nm) primarily shows compounds such as chlorogenic acid, hydroxycinnamic acid derivatives, and cinnamic acid, which absorb at a higher wavelength, indicating that these are more conjugated phenolic compounds. The shared peak at 3.967 min in both figures suggests that quercetin is present and has significant absorbance at both wavelengths, which is typical for flavonoids with conjugated double bonds. The distinct retention times and different compounds detected at varying wavelengths underscore the complementary nature of UV detection in identifying and characterizing various polyphenolic compounds.

**FIGURE 2:**
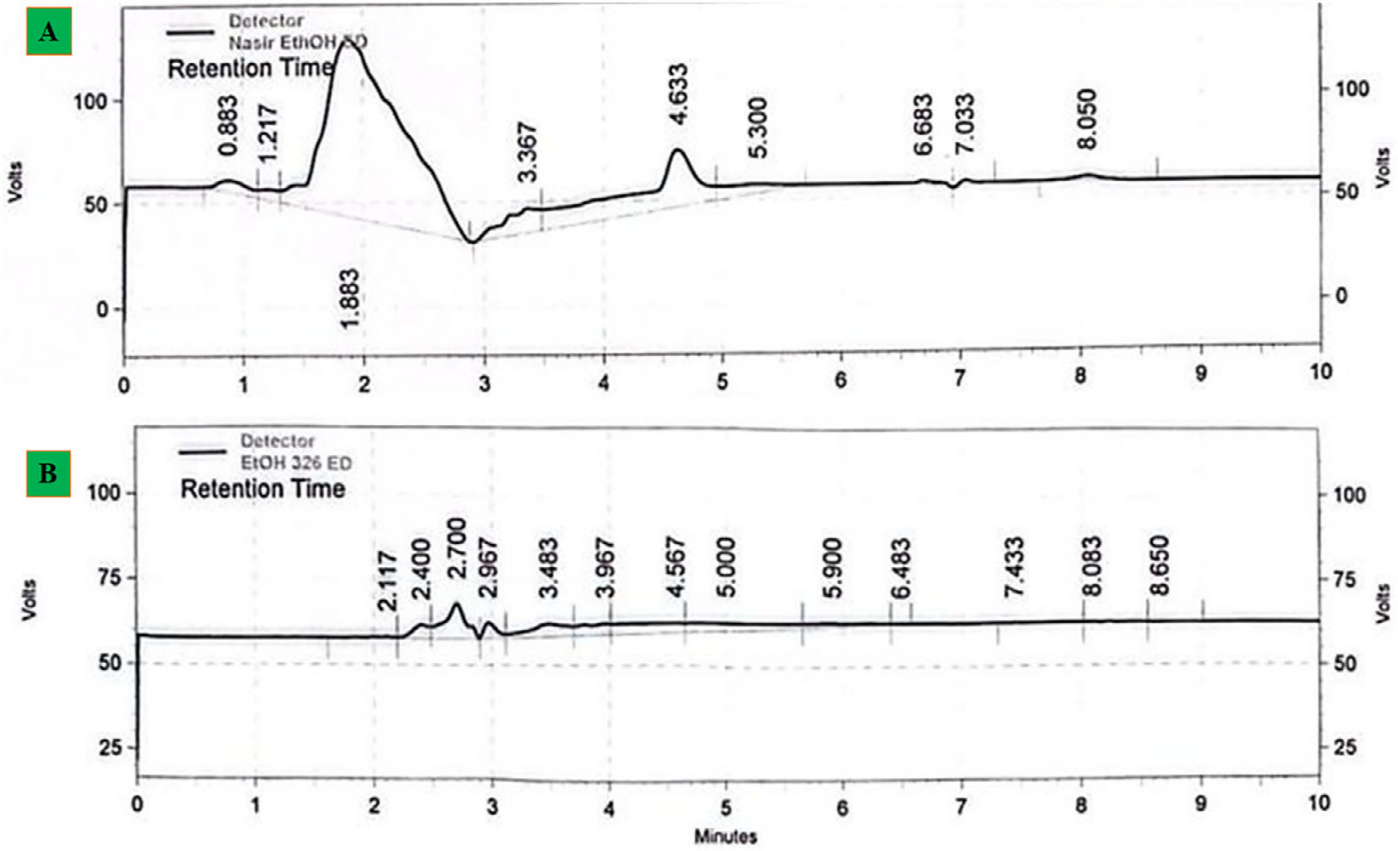
HPLC chromatograms showing the retention profiles of *E. diffusum* ethanol extracts at 254 and 326 nm. Peaks indicate the presence of various phytochemical constituents based on their retention times, highlighting differences in compound composition between the standard and plant extract.

Phenolic acids and flavonoid derivatives (Rt, 1.217 min) compounds typically absorb strongly at lower UV wavelengths, such as 254 nm. According to literature, compounds like gallic acid and other small phenolic acids have retention times in the lower range (1–2 min) and are detected well at 254 nm [26, 27]. Flavonoid glycosides, which are also present in many plant extracts, may elute early under certain chromatographic conditions. Chlorogenic acid, (Rt 2.117 min) a well‐known hydroxycinnamic acid derivative, has a strong absorbance at higher wavelengths, particularly around 326 nm. This is supported by findings in various studies that report its detection in the 2–3 min range in reversed‐phase HPLC analyses [28]. Quercetin/Kaempferol and Hydroxycinnamic Acid Derivatives (Rt 3.967 min, shared peak at 254 and 326 nm), which are flavonoids, typically exhibit significant absorbance at both 254 and 326 nm due to their conjugated systems. This is consistent with literature where quercetin has been shown to elute between 3–5 min in various HPLC protocols, with strong absorbance at both wavelengths [29]. The shared peak at 3.967 min suggests the presence of quercetin, which matches reports from similar studies. Catechins, Derivatives (Rt 4.633 min) another class of flavonoids, often show strong absorbance at 254 nm and elute in the 4–5 min range in HPLC studies. Literature reports suggest that catechin and its derivatives typically elute later than simple phenolic acids due to their larger, more complex structures [30]. Tannins, (Rt 5.300 min) being larger polyphenolic compounds, usually elute last in the chromatographic run. Their detection at 254 nm aligns with literature that discusses the broad absorbance spectrum of these compounds due to their polymeric nature [31]. Cinnamic Acid (its derivatives (Rt 4.567 min) typically absorb at higher wavelengths (around 326 nm) due to the extended conjugation in their structures. Studies report that these compounds elute in the mid‐to‐late range of HPLC analysis.

### 2.4 Detection of the Bioactive Compound Using GC‐MS Analysis

GC‐MS analysis of the ethanolic extract of the ED plant revealed several key bioactive compounds, predominantly oleic acid, palmitic acid, and stearic acid, as shown in Figure 3. The GC‐MS analysis of the ethanol sample revealed a diverse range of compounds, with the most prominent being 2,4‐Di‐*tert*‐butylphenol and 7,9‐Di‐*tert*‐butyl‐1‐oxaspiro [4, 5] deca‐6,9‐diene‐2,8‐dione Bis(2‐ethylhexyl), which accounted for 72.32%. Other significant compounds detected include oleic Acid (11.00%) and 9‐octadecenoic acid methyl ester (4.97%), both of which are fatty acid derivatives. The presence of these fatty acids, along with smaller amounts of hexadecanoic acid and its methyl ester, suggests that the sample may contain lipophilic substances. Additionally, 2,4‐di‐*tert*‐butylphenol and phthalate were identified, as shown in Table 2. Oleic acid, a monounsaturated omega‐9 fatty acid, is known for its cardiovascular benefits, anti‐inflammatory properties, and significant role in skin hydration. Palmitic acid, a saturated fatty acid, is crucial for energy storage and skin protection, whereas stearic acid, another saturated fatty acid, is valued for its stability in skincare formulations. The presence of these fatty acids suggests that ED holds considerable potential for application in nutraceuticals, functional foods, and cosmetics, particularly in products aimed at cardiovascular health, skin protection, and anti‐aging.

**FIGURE 3:**
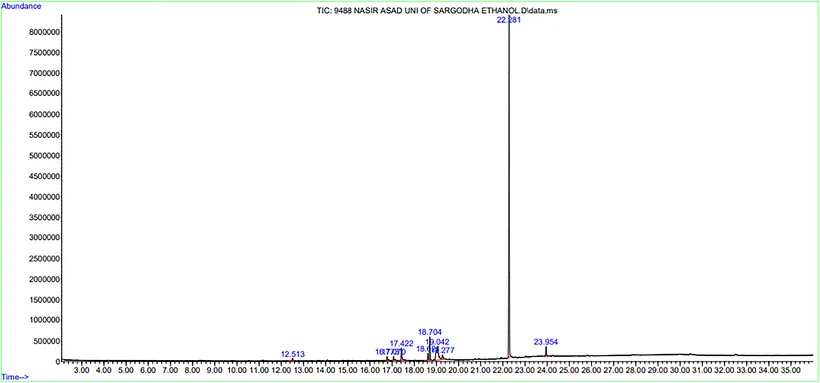
The major volatile phytochemicals detected in the *E. diffusum* ethanol extract manifested in a GC‐MS total ion chromatogram.

**TABLE 2:** Compounds identified from the GC–MS analysis of *E. diffusum*.

S.No	Retention time (min)	Compound name	CAS number	Peak area	Area%
1	12.513	2,4‐Di‐*tert*‐butylphenol	96‐76‐4	1,090,462	0.54
2	16.773	Bis(2‐ethylhexyl) phthalate	117‐81‐7	1,722,225	0.85
3	17.070	Hexadecanoic acid methyl ester	112‐39‐0	1,546,457	0.76
4	17.422	n‐Hexadecanoic acid	57‐10‐3	6,745,997	3.34
5	18.619	10,13‐Octadecadienoic acid methyl ester	56554‐62‐2	3,154,064	1.56
6	18.704	9‐Octadecenoic acid methyl ester	2462‐84‐2	10,057,523	4.97
7	19.042	Oleic acid	112‐80‐1	22,241,586	11.00
8	19.277	Octadecanoic acid	57‐11‐4	3,972,153	1.96
9	22.281	7,9‐Di‐*tert*‐butyl‐1‐oxaspiro(4,5)deca‐6,9‐diene‐2,8‐dione	82304‐66‐3	146,241,457	72.32
10	23.954	1,4‐Benzenedicarboxylic acid bis(2‐ethylhexyl) ester	6422‐86‐2	5,434,035	2.69

A study of the ethanolic leaf extract of *Hellenia speciosa* also showed oleic acid as the main component, along with palmitic acid, which correlates with the ED plant extract. The study indicates the possibilities of these fatty acids in the field of skincare and anti‐inflammatory use [32]​. Phytochemical screening of *Curcuma angustifolia* concluded the existence of palmitic and stearic acids, making them very important in plant‐extracted products as they are naturally protective and moisturizing [33]. In *Piper nigrum* extract both Oleic and palmitic acids were identified in *P. nigrum* extract, indicating their antioxidative and anti‐inflammatory activities, similar to those in ED plants [34]. The oleic, palmitic, and stearic acids of bioactive compounds present in the ethanolic extract of ED not only play a vital role in health and skin care but also possess antimicrobial activity. It contains various fatty acids that have shown antibacterial activity to varying degrees and are hence valuable in the development of antimicrobial agents. Palmitic acid is regularly added to skincare products, apart from its emollient properties, due to its antibacterial properties to prevent and treat bacterial infections. It is also used as a natural antibacterial agent in food preservation [35].

Stearic acid is widely used in cosmetics as a stabilizer, thickener, and antibacterial agent in pharmaceutical formulations. It is particularly applicable to products used for the treatment of bacterial skin infections and as preservatives in food and cosmetics [36]. *Rhazya stricta* was investigated, and it was observed that the plant's fatty acids, including oleic and palmitic acids, were responsible for its antibacterial action against common skin bacteria [37]. *Pistia stratiotes* ethanol extract contains palmitic and oleic acids, both of which are responsible for the broad‐spectrum antibacterial activity, making it an option for developing new antimicrobial [35]. Oleic, palmitic, and stearic acids found in the ED plant extract have been well‐documented as potent antibacterial agents. This finding is consistent with other studies, indicating that ED plants could be potentially employed in the production of natural antibacterial agents for widespread application in the pharmaceutical industry, as well as antioxidant in the cosmetic and food sectors.

### 2.5 Antibacterial Activity

​The dataset presents inhibition zone diameters (in millimeters) for various bacterial strains under three conditions: (a) 30 µg/mL a positive control (b), 20 µg/mL of ED sample (c), and 30 µg/mL of ED extract. The bacterial strains tested included *Staphylococcus epidermidis*, *Listeria monocytogenes*, *Staphylococcus aureus*, *Pseudomonas aeruginosa*, and *Bordetella bronchiseptica* as shown in Figure 4. ​ The inhibition zones for the positive control (a) ranged from 28 to 36 mm, indicating effective antibacterial activity. The 20 µL ED sample (b) shows inhibition zones between 26 and 33 mm, while the 30 µL ED extract (c) exhibits zones from 28 to 37 mm. Notably, the 30 µL ED extract generally produced larger inhibition zones than the 20 µL sample, suggesting a dose‐dependent antibacterial effect, as shown in Figure 5. ​ Statistical analysis using one‐way ANOVA followed by Tukey's post‐hoc test confirmed that the differences in the zones of inhibition between the extract concentrations and the positive control were statistically significant (*p* < 0.05) as shown in Table 3.

**FIGURE 4:**
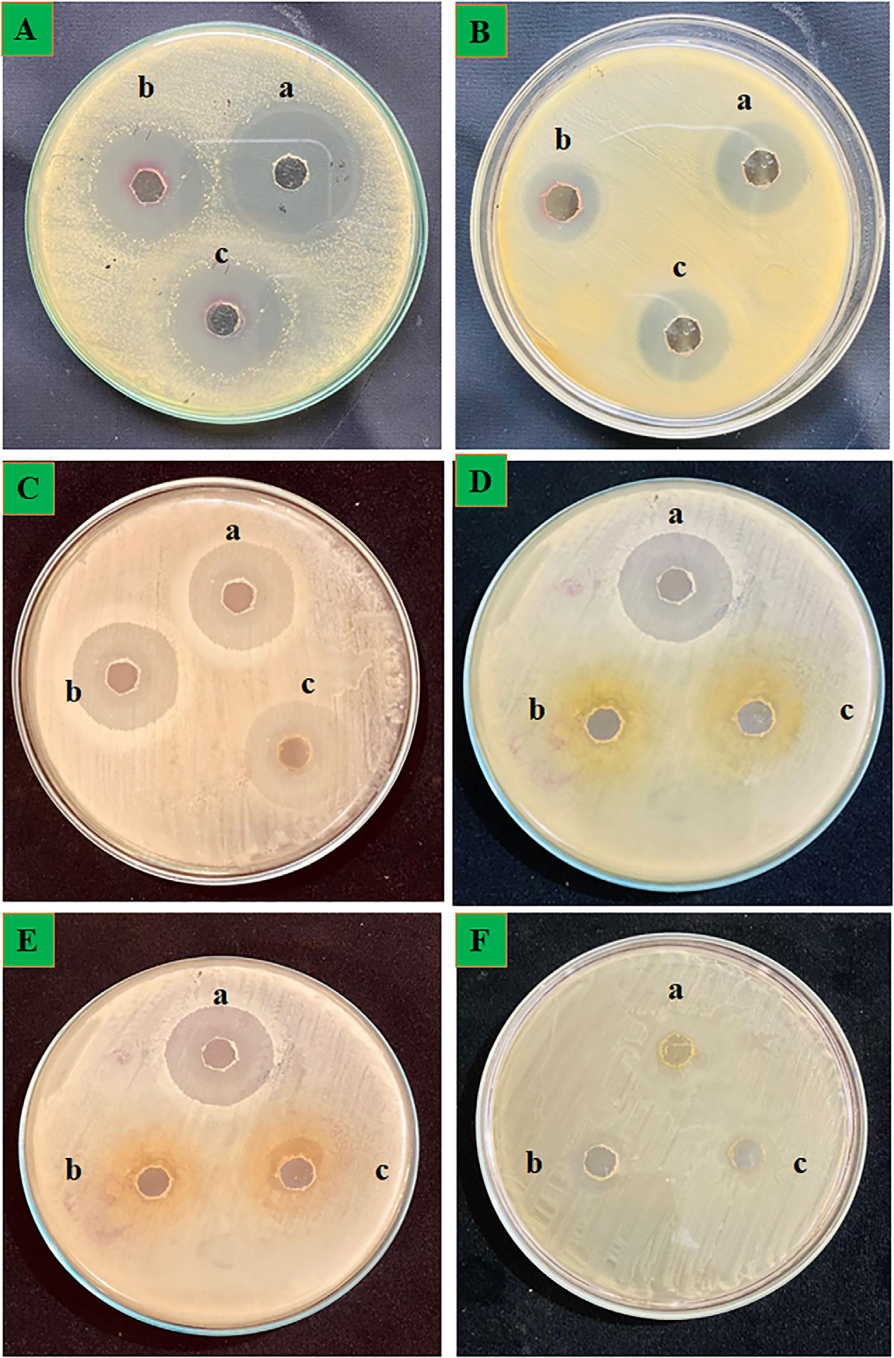
Antibacterial activity against; (A) *S. epidermidis* (ATCC 12228), (B) *L. monocytogenes* (ATCC 13932), (C) *S. aureus (ATTC 9144)*, (D) *P. aeruginosa* (ATCC 10145), (E) *B. bronchiseptica* (ATTC 4617), and (F) *E. coli* (ATCC 10536), using the well diffusion method.

**FIGURE 5:**
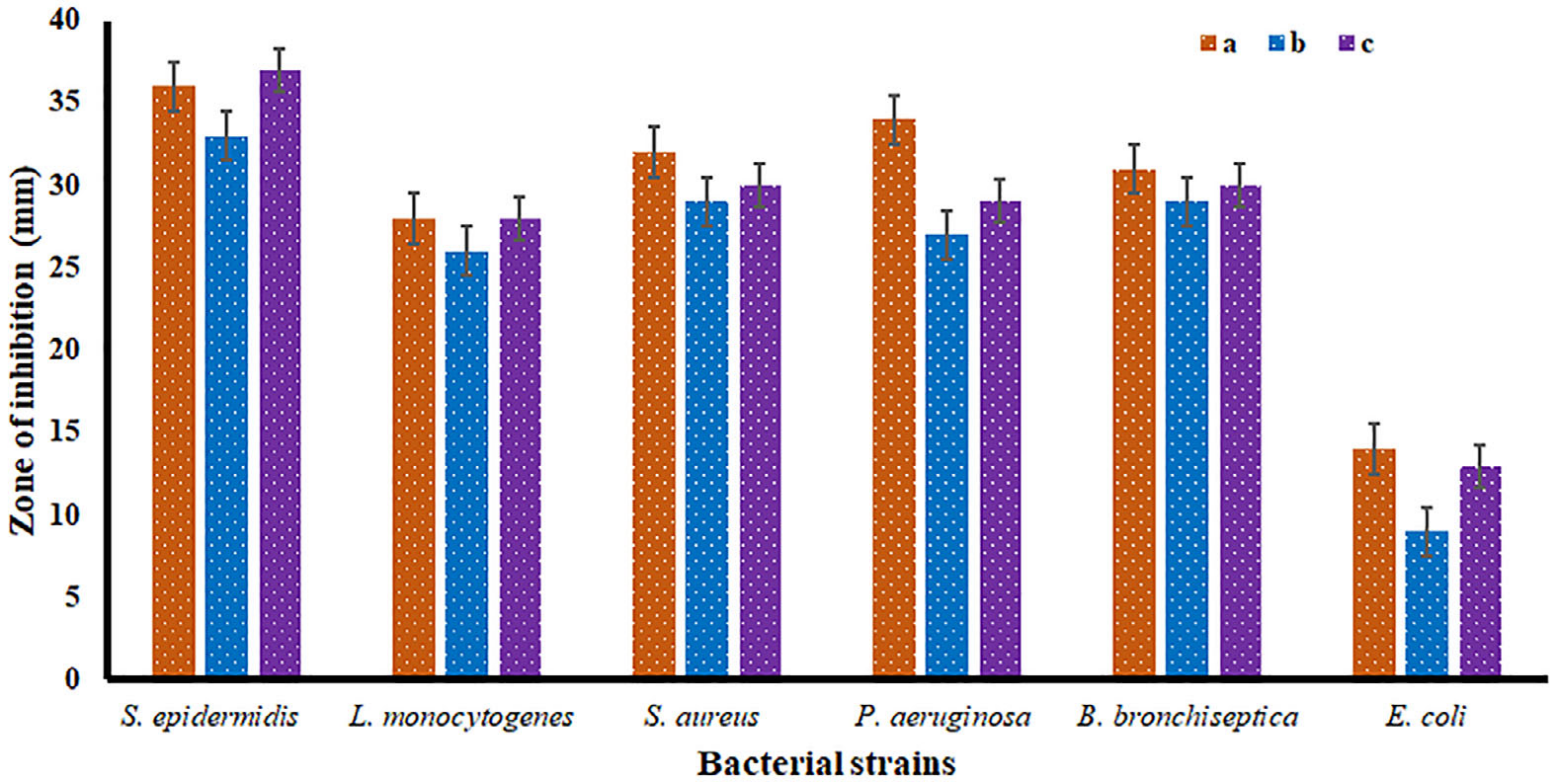
Antibacterial activity of against bacterial strains. Bars display results for (a) the positive control Erythromycin at 30 µg/mL, (b) plant extract at 20 µg/mL, and (c) plant extract at 30 µg/mL. Zone of inhibition (mm) indicates effectiveness, with varying susceptibility observed among different strains.

**TABLE 3:** Antibacterial activity (ZOI in mm) of *E. diffusum* against bacterial strains.

Bacterial strain	Positive control (30 µg/mL)	Extract (20 µg/mL)	Extract (30 µg/mL)
*S. epidermidis*	36.0 ± 0.3^a^	33.0 ± 0.5^b^	37.0 ± 0.6^c^
*L. monocytogenes*	28.0 ± 0.3^a^	26.0 ± 0.3^b^	28.0 ± 0.5^a^
*S. aureus*	32.0 ± 0.5^a^	29.0 ± 0.5^b^	30.0 ± 0.4^c^
*P. aeruginosa*	34.0 ± 0.5^a^	27.0 ± 0.5^b^	29.0 ± 0.5^c^
*B. bronchiseptica*	31.0 ± 0.5^a^	29.0 ± 0.5^b^	30.0 ± 0.5^c^
*E. coli*	14.0 ± 0.5^a^	9.0 ± 0.5^b^	13.0 ± 0.5^c^

*Note*: Data is presented as Mean ± SD. Letters (a, b, c) denote statistical significance within each row: values not sharing the same letter are significantly different at *p* < 0.05.

Plant extracts exert antibacterial effects via various mechanisms [38]. One primary mode of action is the disruption of bacterial cell membranes, leading to increased permeability and leakage of cellular content [39]. Flavonoids, a class of polyphenolic compounds found in many plants, have been shown to inhibit bacterial growth by binding to bacterial enzymes via hydrogen bonds, inducing modifications in cell membrane permeability and cell wall integrity [40]. ​Additionally, plant‐derived compounds can interfere with bacterial enzyme activity, inhibit nucleic acid synthesis, and disrupt energy metabolism. For instance, certain flavonoids have been reported to inhibit DNA gyrase, an essential enzyme involved in bacterial DNA replication [41]. Furthermore, phenolic compounds can act as efflux pump inhibitors, reducing the ability of bacteria to expel antimicrobial agents, thereby enhancing their susceptibility [42]. ​

The ethanolic extract of *E. diffusum* was evaluated for its MIC and MBC activity against six bacterial strains. The results are presented in Table 4. These findings suggest that the ED extract has significant antibacterial activity, particularly against Gram‐positive bacteria. *S. epidermidis* showed the highest susceptibility, with a zone of inhibition (ZOI) of 37.0 ± 0.6 mm at 30 µg/mL and MIC and MBC values of 30 ± 0.4 and 50 ± 0.5 µg/mL, respectively. *L. monocytogenes* also showed high sensitivity with a ZOI of 28.0 ± 0.5 mm at 30 µg/mL and MIC and MBC values of 30 ± 0.3 and 50 ± 0.4 µg/mL, respectively. The extract also showed significant antibacterial activity against *S. aureus* with a ZOI of 30.0 ± 0.4 mm at 30 µg/mL, and MIC and MBC of 40 ± 0.5 and 60 ± 0.5 µg/mL, respectively [43].

**TABLE 4:** *E. diffusum* ethanol extract antibacterial activity, MIC, and MBC against selected bacterial strains. The data are presented as the average ± standard deviation, with a sample size of 3.

Bacterial strain	MIC (µg/mL)	MBC (µg/mL)
*S. epidermidis*	30 ± 0.4	50 ± 0.5
*L. monocytogenes*	30 ± 0.3	50 ± 0.4
*S. aureus*	40 ± 0.5	60 ± 0.5
*P. aeruginosa*	40 ± 0.5	70 ± 0.6
*B. bronchiseptica*	40 ± 0.5	70 ± 0.5
*E. coli*	50 ± 0.3	100 ± 0.6

Moderate susceptibility was observed with *P. aeruginosa* and *B. bronchiseptica*, with ZOIs of 29.0 ± 0.5 and 30.0 ± 0.5 mm at 30 µg/mL, respectively. Both of these strains possessed MICs of 40 ± 0.5 µg/mL and MBCs of 70 ± 0.6 and 70 ± 0.5 µg/mL, respectively. *E. coli* showed the lowest susceptibility with a ZOI of 13.0 ± 0.5 mm at 30 µg/mL and the highest MIC and MBC values of 50 ± 0.3 and 100 ± 0.6 µg/mL, respectively. Our results are inconsistent with those of other studies, suggesting that gram‐negative bacteria are generally more resistant to plant extracts because their outer membrane may act as a permeability barrier.

The higher activity against gram‐positive bacteria might be explained by the phytochemical compounds of *E. diffusum*, flavonoids, and phenolic acids, which are known to have antimicrobial properties. Further phytochemical studies and identification of bioactive compounds need to be conducted to characterize the exact compounds responsible for the observed antibacterial activities. Therefore, the ethanolic extract of *E. diffusum* exhibited substantial bacterial growth inhibitory effects, especially on gram‐positive bacteria, indicating its potential as a natural antimicrobial compound [44].

### 2.6 Antioxidant Activity

#### 2.6.1 Diphenyl‐1‐picrylhydrazyl (DPPH) Assay

The DPPH (2,2‐diphenyl‐1‐picrylhydrazyl) assay is a widely used method to evaluate the free radical scavenging ability of antioxidants, where the DPPH radical, exhibiting a deep violet color, absorbs at 517 nm and decolorizes upon reduction by an antioxidant. Statistical analysis using two‐way ANOVA revealed a significant effect of treatment group and concentration on DPPH radical scavenging activity (*p* < 0.05). The plant extract showed a significant dose‐dependent increase in %RSA, while the standard antioxidant exhibited significantly higher but concentration‐independent activity. A differential response pattern between plant extract and standard was observed across concentrations. The ED extract shows a concentration‐dependent increase in scavenging activity from 48.51% at 100 µg/mL to 69.7% at 500 µg/mL, while ascorbic acid, a well‐known antioxidant, showed scavenging activity of 73.57% at 500 µg/mL concentration shown in Figure 6. This indicates that the ED extract has notable antioxidant potential, and the scavenging efficiency was comparable to that of ascorbic acid, suggesting effective potency as ascorbic acid in neutralizing free radicals [45, 46]. Thus, observed concentration‐dependent radical scavenging efficiency validates the efficacy of* E*. *diffusum* as a natural scavenger of free radicals.

**FIGURE 6:**
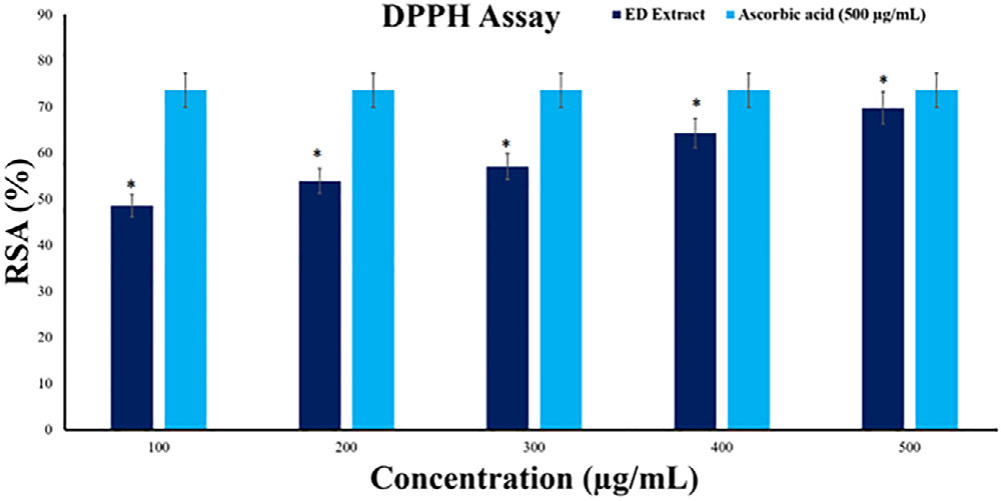
DPPH radical scavenging activity (RSA%) of *E. diffusum* extract. Values are expressed as mean ± standard digits (*n* = 3). Significant differences between concentrations were determined via two‐way ANOVA followed by Tukey's post‐hoc test (*p* < 0.05). Asterisks (*) denote significant differences compared to the 100 µg/mL concentration group.

The antioxidant property of the extract of *E. diffusum*, as indicated by the DPPH radical scavenging activity, increased in a concentration dependent manner which is similar to that reported for the other Equisetum species in recent studies. The antioxidant activity of *Equisetum ramosissimum* stem extracts was remarkable in DPPH and other antioxidant assays, while their total phenolic and flavonoid content was high [47]. In addition, comparative phytochemical analysis of several species of Equisetum confirmed that flavonoids, hydroxycinnamic, and hydroxybenzoic acids are present in all species and are known to have DPPH scavenging activity in comparative study [22]. The results corroborate the notion that the antioxidant effect of *E. diffusum*, like other species in the genus, can be attributed to a mixture of phenolic compounds. The activities are in line with ethnopharmacological findings that indicate Equisetum extracts as potential sources of natural antioxidants with health‐promoting properties [48].

#### 2.6.2 FRAP Assay

The present study evaluated the antioxidant potential of *E. diffusum* extract using the FRAP assay, which measures the ability of antioxidants to convert Fe^3^^+^ to Fe^2^^+^. The results demonstrated a dose‐dependent increase in reducing power, with the ED extract exhibiting 27.03% inhibition at 100 µg/mL, which progressively increased to 70% at 500 µg/mL. In comparison, ascorbic acid (500 µg/mL) showed inhibition of 77.03% across at 500 µg/mL, highlighting its potent antioxidant capacity, as shown in in Figure 7. Statistical analysis using two‐way ANOVA indicated a significant effect of the treatment group and concentration on FRAP activity (*p* < 0.05). The plant extract exhibited a significant concentration‐dependent increase in reducing activity, whereas the standard antioxidant remained relatively constant across the tested concentrations. Although the ED extract showed significant ferric‐reducing potential, its activity remained comparable to that of ascorbic acid, suggesting the presence of bioactive compounds contributing to its antioxidant properties.

**FIGURE 7:**
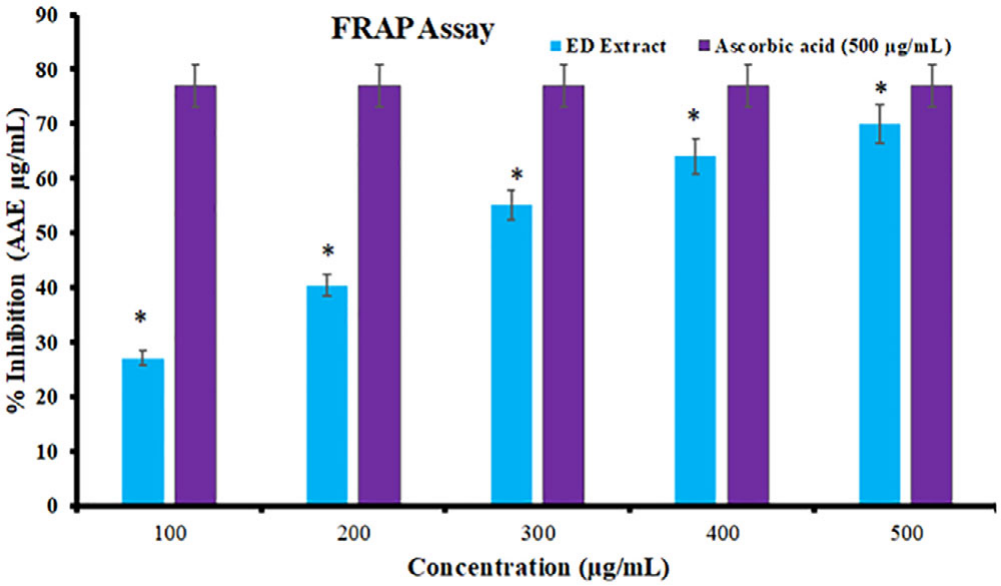
FRAP assay of *E. diffusum* extract. Values are expressed as mean ± standard digits (*n* = 3). Values are expressed as mean ± SD (*n* = 3). Statistical significance was determined using two‐way ANOVA followed by Tukey's post‐hoc test (*p* < 0.05). Significant differences between the extract and standard at each concentration are indicated by asterisks (*).

The dose‑dependent *increase in ferric‑reducing antioxidant power demonstrated by the E. diffusum* extract is supported by recent studies on related horsetail species. The antioxidant activity was significant in ethanol extracts of *Equisetum arvense* L. using various assays, with the FRAP assay, and the amounts of phenolics and flavonoids measured were high [49]. The observations suggest that antioxidants from horsetail extracts may donate electrons to the reduction of Fe^3^^+^ to Fe^2^^+^, which is a major antioxidant mechanism. Increasing the activity of reducing power with the increase of concentration of plant extract in antioxidant studies, which is close to the activity of ascorbic acid, is a common finding that could be attributed to the synergistic action between more than one bioactive compound. The robust reducing activity of *E. diffusum* extract thus highlights its promise as a natural antioxidant source suitable for further exploration in functional food and pharmaceutical applications.

#### 2.6.3 Total Flavonoid Content Assay

The Total Flavonoid Content (TFC) assay was conducted to quantify the flavonoid content of the ED extract, using quercetin as the standard. The results demonstrated a concentration‐dependent increase in flavonoid content, with the ED extract exhibiting 37.18% inhibition at 100 µg/mL, progressively increased to 76.4% at 500 µg/mL. In comparison, quercetin (500 µg/mL) consistently exhibited 80.5% inhibition 500 µg/mL. Statistical analysis using two‐way ANOVA confirmed that the variation in TFC between the extract and the standard, as well as the concentration‐dependent increase, were statistically significant (*p* < 0.05). As illustrated in Figure 8, the extract maintained a lower inhibition profile compared to the quercetin standard across all concentrations, with significant differences marked by asterisks (*).

**FIGURE 8:**
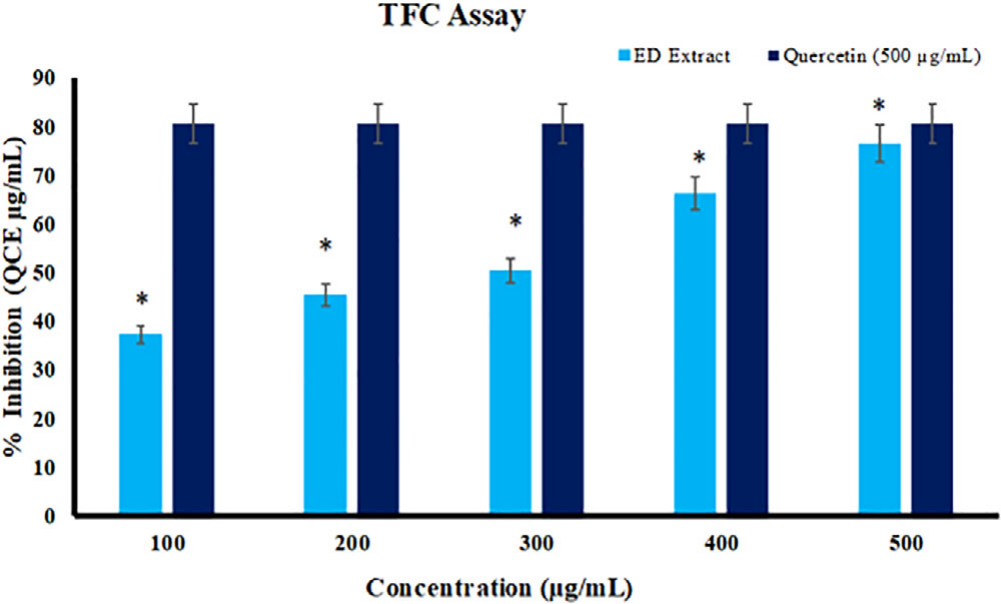
TFC assay of *E. diffusum* extract. Values are expressed as mean ± standard digits (*n* = 3). Values are expressed as mean ± SD (*n* = 3). Statistical significance was determined using two‐way ANOVA followed by Tukey's post‐hoc test (*p* < 0.05).

Although the ED extract showed significant flavonoid accumulation, it remained slightly lower than that of the quercetin standard. These findings suggest that the ED extract possesses substantial flavonoid content, contributing to its potential antioxidant and pharmacological properties, suggesting further investigation into its bioactive compounds and therapeutic applications.

### 2.7 In Silico Analysis

#### 2.7.1 Molecular Interaction Analysis

All studied phytochemicals (about 10) of GC‐MS were observed for molecular docking with antioxidant and antimicrobial proteins in order to observed which mainly ligand was responsible for these studied. All phytochemicals toxicity prediction were done by ProTox‐11 web server [50]. The examination and documentation of structural compounds derived from various medicinal plant species are increasingly recognized for their significance and relevance. In structure‐based drug design, molecular docking is typically employed to predict and analyze the intermolecular complex between compounds and their target proteins [51]. The findings from the molecular docking of different ligands with the antimicrobial protein (1R4U) and antioxidant protein (1AJ6) are detailed in Table 6. The table lists each ligand's chemical name, binding strength (in kcal/mol), interaction sites, and length (in Å). Binding affinity reflects the strength of the interaction between the ligand and protein. A reduced Δ*G* value indicates a stronger binding affinity between the ligand and protein [52]. The results have been interpreted by BIOVIA Discovery Studio which revealed that Bis(2‐ethylhexyl) phthalate (ligand 5) have shown the high binding score of −6.6 kcal/mol and interacted via THR74, TYR30, MET32, CYS103, PRO76, ARG128, ARG105, and TRP208 to antimicrobial protein but it has shown slightly active in carcinogrencit 5. Ligand 1 interacting residues were found to be TRP106, VAL29, TYR30, CYS103, and PRO76. Ligand 2 interacted via TRP208, TRP208, and GLU31 and ligand 3 interacted via TYR30, CYS103, PRO76, TRP208, TRP106, and THR107. Ligand 4 interacted with ARG105, TYR30, CYS103, TRP208, ARG128, and GLU126 show best binding results with both target proteins. The interacting residues for ligand 6 were, ARG105, CYS103, and TYR30. Ligand 7 interacted via ARG128, TRP208, ARG105, and GLU31. Ligand 8 interacted through CYS103, PRO76, TRP208, and GLU126. Binding site interaction residues for Ligand 9 were, TYR30, CYS103, PRO76, ARG128, TRP208, ARG105, TRP106, and THR107. Ligand 10 interacted with the protein through TYR30, CYS103, ARG128, ASP205, THR74, ARG105, and TRP208.

For the antioxidant protein, bis (2‐ethylhexyl) terephthalate (ligand 6) showed the best binding affinity of −6.5 kcal/mol and interacted via VAL71, VAL43, ALA47, ASP73, THR165, GLY77, GLU50, ARG76, ILE94, PRO79, ILE78, and ALA90. However, it was active in carcinogenicity, as shown in Table 4. The ligand 1 binding interactions were shown by ILE78 and VAL167. Ligand 2 interacted through ILE78, ILE94, and GLY77, and ligand 3 via HIS99, ILE94, ILE78, and GLU50. The interacting residues of ligand 4 were ILE78 and ILE94, and ligand 5 interacted through ILE94, ILE78, ASN46, ALA47, VAL43, VAL167, and VAL71. Ligand 7 interacted via ILE94, VAL43, ASN46, and ILE78. The binding interaction residues of ligand 8 were VAL167, THR165, VAL43, ALA47, VAL71, ILE78, ILE94, and GLY119. Ligand 9 interacted with PRO79, GLY77, ASP73, THR165, ALA47, VAL167, VAL43, VAL71, ILE78, and ILE94. Ligand 10 interacted with the protein through ILE78, ILE94, ASN46, and VAL120. These results are consistent with those of previous studies [53, 54] (Table 5).

**TABLE 5:** Pro Tox II prediction of phytochemicals of *E. diffusum* (LD_50_, Toxicity class).

Phytochemicals	LD_50_mg/kg	Class	Mutagencity	Hepatoxcity	Immuno ‐ toxicity	Cytotoxcity	Carcinogenicity
Oleic acid	48	2	Inactive	Inactive	Inactive	Inactive	Inactive
n‐Hexadecanoicacid	900	4	Inactive	Inactive	Inactive	Inactive	Inactive
Methyl elaidate	3000	5	Inactive	Inactive	Inactive	Inactive	Inactive
hexadecanoic acid methyl ester	000	5	Inactive	Inactive	Inactive	Inactive	Inactive
Bis(2‐ethylhexyl) terephthalate	3200	5	Inactive	Inactive	Inactive	Inactive	Active
Bis(2‐ethylhexyl) phthalate	1340	4	Inactive	Inactive	Inactive	Inactive	Acive
10,13‐Octadecadienoic acid, methyl ester	20,000	6	Inactive	Inactive	Inactive	Inactive	Inactive
9‐octadecenoic acid methyl ester	300	5	Inactive	Inactive	Inactive	Inactive	Inactive
7,9‐Di‐*tert*‐butyl‐1‐oxaspiro(4,5)deca‐6,9‐diene‐2,8‐dione	900	4	Inactive	Inactive	Inactive	Inactive	Inactive
2 4‐di‐*tert*‐butylphenol	1190	4	Inactive	Inactive	Active	Inactive	Inactive

*Note*: The color coding in the toxicity predictions corresponds to the ProTox‐II server output: dark red indicates high‐probability (“Active”); pink indicates borderline/low‐probability (“Active”); dark green indicates high‐confidence (“Inactive”); and light green indicates borderline (“Inactive”).

Among the various phytochemicals analyzed, 7,9‐di‐*tert*‐butyl‐1‐oxaspiro(4,5)deca‐6,9‐diene‐2,8‐dione exhibited a notable binding affinity of −7.4 and −7.3 kcal/mol, indicating strong molecular interactions with the target protein (TA), suggesting that its main activity is driven by these ligands (Table 6). It satisfies Lipinski's Rule of Five with no violations, ensuring good oral bioavailability, and passes all major drug‐likeness filters (Ghose, Veber, Egan, and Muegge), confirming its suitability as a drug candidate [55], as shown in Figure 9. The favorable binding characteristics, stability, and molecular properties of this compound position it as a promising lead for drug development, particularly in antimicrobial and antioxidant applications. Further in vitro and in vivo investigations are necessary to evaluate the pharmacological efficacy, toxicity, and formulation potential of this compound.

**TABLE 6:** Docking scores and residues involved in the H‐bond formation of the selected compounds.

		1R4U (anti‐microbial protein)		1AJ6 (antioxidant protein)	
Ligand Code	Chemical name	Binding energy Δ*G* (kcal/mol)	Binding site interaction residues and distance (Å)	Binding energy Δ*G* (kcal/mol)	Binding site interaction residues and distance (Å)
1	2 4‐di‐*tert* butylphenol	−5.9	TRP106 (5.05) VAL29 (5.56) TYR30 (4.78) CYS103 (5.44) CYS103 (6.25) PRO76 (5.32)	−6.0	ILE78 (5.04) ILE78 (5.77) VAL167 (4.99)
2	7,9‐Di‐*tert*‐butyl‐1‐oxaspiro(4,5)deca‐6,9‐diene‐2,8‐dione	−7.3	TRP208 (4.93) TRP208 (6.19) GLU31 (4.97)	−7.1	ILE78 (5.25) ILE94 (5.06) GLY77 (3.39)
3	9‐Octadecenoic acid methyl ester	−4.5	TYR30 (5.42) TYR30 (5.23) TYR30 (4.35) CYS103 (4.68) CYS103 (5.89) PRO76 (6.36) TRP208 (6.52) TRP106 (6.50) THR107 (4.19)	−5.4	HIS99 (5.17) ILE94 (5.11) ILE78 (6.69) ILE78 (4.05) GLU50 (5.22) GLU50 (4.26)
4	10,13‐Octadecadienoic acid, methyl ester	−5.0	ARG105 (5.11) TYR30 (4.99) TYR30 (6.14) CYS103 (6.58) TRP208 (5.04) ARG128 (5.08) GLU126 (4.11)	−4.8	ILE78 (5.47) ILE78 (3.96) ILE94 (5.76)
5	Bis(2‐ethylhexyl) phthalate	−6.6	THR74 (4.37) TYR30 (5.02) MET32 (6.41) CYS103 (4.34) PRO76 (6.38) PRO76 (4.75) ARG128 (3.81) ARG128 (5.07) ARG105 (5.49) ARG105 (4.21) TRP208 (6.95) TRP208 (6.13) TRP208 (7.43)	−5.7	ILE94 (4.89) ILE78 (4.97) ASN46 (4.37) ASN46 (4.13) ALA47 (5.65) ALA47 (4.67) VAL43 (4.86) VAL167 (4.86) VAL71 (4.23)
6	Bis(2‐ethylhexyl) terephthalate	−5.6	ARG105 (4.12) CYS103 (4.37) TYR30 (6.46) TYR30 (4.45)	−6.5	VAL71 (3.99) VAL43 (4.93) ALA47 (4.54) ALA47 (5.37) ASP73 (5.93) THR165 (4.18) GLY77 (3.40) GLU50 (4.91) ARG76 (4.71) ARG76 (5.31) ILE94 (4.90) PRO79 (7.14) PRO79 (4.90) ILE78 (5.07) ILE78 (3.96) ILE78 (4.97) ALA90 (6.29)
7	Hexadecanoic acid methyl ester	−4.4	ARG128 (5.24) TRP208 (7.06) TRP208 (5.33) ARG105 (4.39) ARG105 (4.35) GLU31 (5.02) GLU31 (5.61)	−4.8	ILE94 (4.37) VAL43 (4.34) ASN46 (3.92) ILE78 (4.64) ILE78 (5.77) ILE78 (4.06)
8	Methyl elaidate	−4.6	CYS103 (7.32) PRO76 (6.74) TRP208 (4.90) GLU126 (4.41)	−5.2	VAL167 (5.62) THR165 (5.66) VAL43 (5.10) ALA47 (4.65) VAL71 (4.12) ILE78 (4.92) ILE78 (4.52) ILE78 (5.62) ILE94 (4.37) GLY119 (4.12)
9	n‐Hexadecanoic acid	−4.5	TYR30 (4.96) TYR30 (4.86) CYS103 (5.91) CYS103 (5.22) PRO76 (6.71) ARG128 (3.91) ARG128 (7.00) TRP208 (7.20) ARG105 (3.95) TRP106 (6.49) THR107 (4.07) THR107 (3.51)	−5.2	PRO79 (6.15) GLY77 (3.15) ASP73 (5.23) THR165 (4.19) ALA47 (5.98) VAL167 (4.21) VAL43 (5.28) VAL71 (4.45) ILE78 (5.63) ILE78 (3.79) ILE78 (4.16) ILE94 (5.62)
10	Oleic acid	−5.0	TYR30 (5.00) CYS103 (5.43) ARG128 (3.66) ARG128 (5.00) ARG128 (5.32) ASP205 (5.24) THR74 (4.80) ARG105 (6.25) TRP208 (5.46)	−5.2	ILE78 (5.77) ILE78 (3.70) ILE78 (6.76) ILE94 (5.00) ASN46 (5.26) VAL120 (4.03)

**FIGURE 9:**
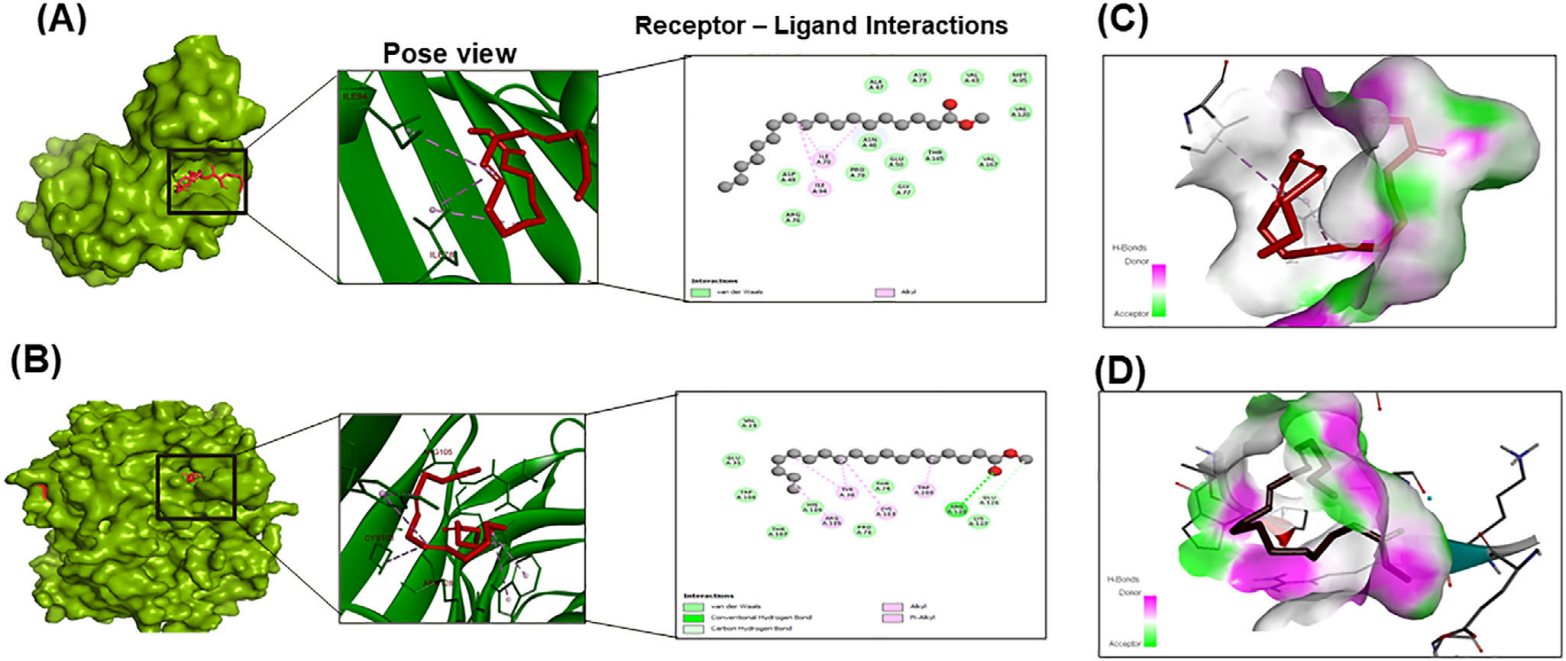
(A) Different binding pose of, 7,9‐di‐*tert*‐butyl‐1‐oxaspiro [4, 5] deca‐6,9‐diene‐2,8‐dione with the anti‐microbial protein (PDB ID: 1R4U) and antioxidant protein (PDB ID: 1AJ6) (B) while hydrophobic interactions were indicated by both, 7,9‐di‐*tert*‐butyl‐1‐oxaspiro [4, 5] deca‐6,9‐diene‐2,8‐dione complex proteins, purple area show Hydrogen donor area and green hydrogen acceptor area (C, D) respectively.

Except for bis(2‐ethylhexyl) terephthalate, bis (2‐ethylhexyl) phthalate, and 2,4‐di‐*tert*‐butylphenol, all other tested phytochemicals exhibited no mutagenicity, hepatotoxicity, immunotoxicity, cytotoxicity, or carcinogenicity, indicating their potential safety for pharmaceutical applications. However, further studies are required to confirm the toxic effects of these three compounds before considering their therapeutic potentials.

Van der Waals and hydrogen bonds are the most prevalent interactions in molecular docking studies. Additional interactions, including π–π, π‐alkyl, and alkyl interactions were also noted. The observed differences in the bonds produced during contact can be attributed to the varying amino acid composition of each enzyme and the accessibility of the amino acids. Enzymes may alter the three‐dimensional structure of the examined chemicals through their interactions with them [56].

## 3 Conclusion

Medicinal plants have long served as reservoirs of therapeutic compounds, offering natural alternatives for combating microbial infections and oxidative stress. *E. diffusum* a lesser‐explored Himalayan species, was investigated for its bioactive potential using integrative analytical approaches. Phylogenetic analysis confirmed its evolutionary linkage within Group C of the *Equisetum* genus, closely related to *E. ramosissimum subsp. debile*, supporting the conserved nature of its target proteins. Phytochemical screening of various solvent extracts revealed the presence of key secondary metabolites such as alkaloids, flavonoids, phenols, and fatty acids, predominantly in the ethanolic extract. The antibacterial evaluation demonstrated broad‐spectrum activity, with the ethanolic extract showing significant inhibition zones (up to 37 mm) against both Gram‐positive and Gram‐negative strains. The MBC values ranged from 50 to 100 µg/mL, confirming the bactericidal nature of the extract against most tested strains. Antioxidant assays (DPPH, FRAP, and TFC) further highlighted its strong radical scavenging and reducing power, with peak activities observed at 500 µg/mL—comparable to standard antioxidants. Molecular docking identified 7,9‐di‐*tert*‐butyl‐1‐oxaspiro [4, 5] deca‐6,9‐diene‐2,8‐dione as the most promising ligand, which showed a high binding affinity (−7.4 and −7.3 kcal/mol) for the antimicrobial and antioxidant target proteins, respectively. This compound also fulfilled the major drug‐likeness and ADME criteria, suggesting the potential for oral bioavailability. Overall, these findings establish *E. diffusum*. as a potent source of antimicrobial and antioxidant agents. Further in vivo studies and formulation work are recommended to translate these insights into therapeutic applications.

## 4 Material and Methods

*E. diffusum* (ED) plants were collected from Mir Ali, North Waziristan, Khyber Pakhtunkhwa, Pakistan. The botanical identification and authentication of the plant specimen were performed by Mrs. Naima Huma Naveed, Assistant Professor, Department of Botany, University of Sargodha, Pakistan. The voucher number (SARGU/CHEM‐027) was assigned to the plant specimen and then deposited in the herbarium (University of Sargodha‐SARGU), Department of Botany, University of Sargodha, Punjab, Pakistan. Plant material was collected from private land belonging to one of the authors, and no specific collection permission was required. All procedures were conducted in accordance with relevant national and international guidelines. All the essential reagents, such as DPPH, Folin–Ciocalteu reagent, ethanol (99.8%), and ascorbic acid (obtained from Sigma‐Aldrich), as well as Mueller–Hinton agar (Oxoid) and cultures of microbial isolates, were provided by the Biochemistry Laboratory, Institute of Chemistry, University of Sargodha. All reagents used in the experimental techniques were of analytical grade, and deionized water (DW) was used to prepare the solutions.

### 4.1 Extraction

Fresh ED. The plant sample was first rinsed with tap water and then with deionized water to remove dust and surface impurities. The cleaned plant material was air‐dried in the shade for 15 days. After complete drying, the material was ground into a fine powder using an electric grinder and then sieved through a 75 µm mesh, as per the modified protocols of the method of Assad et al. (2025) and Rahman et al. (2026) [57, 58]. For extract preparation, 5 g of powdered sample was taken and mixed with 50 mL of ethanol and stirred for 5 h at room temperature. The same method was used for the extraction of aqueous, acetone, n‐hexane, and chloroform extracts. Following extraction, the mixtures were filtered using Whatman No. 42 filter papers, and the recovered filtrates were refrigerated at 4°C for post‐analysis.

### 4.2 Phylogenetic Analysis

Phylogenetic analysis of *Equisetum* species was conducted using molecular sequencing data to assess their evolutionary relationships and taxonomic positions. Total nucleic acid was extracted from plant samples using a standard genomic DNA isolation protocol [59], followed by PCR amplification of the target gene regions. The amplified sequences were aligned and analyzed using the Maximum Likelihood (ML) method, with bootstrap resampling (≥1000 replicates) to ensure statistical robustness. The phylogenetic tree was constructed using MEGA software, with *E. telmateia subsp. braunii* designated as the outgroup for rooting the tree. Sequence comparisons were further validated using the publicly available NCBI GenBank data. The classification of clades was supported by high bootstrap values and genetic distance calculations, ensuring the reliability of the evolutionary relationships among *Equisetum* species. The sequence PV013407, included in Group C, was analyzed and compared with closely related *E. ramosissimum subsp. debile* isolates, confirming its phylogenetic position within this clade.

#### 4.2.1 PCR Amplification and Gel Electrophoresis

PCR amplification and electrophoresis procedures were carried out using standard molecular biology reagents and equipment. DNA was extracted using the Qiagen AMP DNA Mini Kit (Cat# 51306) and quantified with a Nanodrop spectrophotometer. PCR was performed using Thermo Scientific DreamTaq Green Master Mix (Cat# K1081) in an Applied Biosystems GeneAmp PCR System 2700. To determine the size of the amplified DNA fragments, a 100 bp DNA ladder (Thermo Scientific, Cat# SM0323) was used as a molecular weight marker. The PCR was carried out with an initial denaturation at 94°C for 5 min, followed by 35 cycles consisting of denaturation at 94°C for 30 s annealing at 55°C for 30 s, and extension at 72°C for 50 s. The process concluded with a final extension step at 72°C for 5 min. PCR products were separated and visualized using agarose gel electrophoresis. A 1.0% agarose gel was used for fragments greater than 1000 bp, while a 1.5% gel was used for fragments smaller than 500 bp (Figure 2S (Supporting File 1)). All gels were prepared in 1X TAE buffer. Ethidium bromide was used for staining, and gels were run at 110 V for 30 min, then visualized under UV light. Gel‐purified PCR products were extracted using the GeneJET Gel Extraction Kit (Cat# K0691). Sequencing was performed by APICAL SCIENTIFIC SDN. BHD. All materials used, including agarose (Thermo Scientific, Ref# R0491), absolute ethanol (Sigma Aldrich, Cat# KO6300000), and electrophoresis equipment (JY‐SPCT tank, power supply model 500/200, Hoofer MC1400 centrifuge), supported reliable amplification and analysis.

### 4.3 Qualitative Phytochemical Screening

A qualitative phytochemical study of (ED) extracts procured with ethanol, (DW), n‐hexane, chloroform, and acetone was carried out using standardized methods. Alkaloid presence was verified through the formation of a reddish‐brown precipitate when Wagner's reagent was added [60]. Flavonoids were detected by applying the extract with sodium hydroxide, which resulted in the formation of yellow to red, signifying the presence of phenolic compounds [61]. The presence of saponins was established by mixing the extract with an equal volume of DW and centrifuging the mixture for 15 min. The persistence of a foamy layer on the surface indicated the presence of saponins [25]. The presence of phenolic compounds was confirmed by the blue–black color change upon the addition of ferric chloride to the extract [62]. To check for steroids, the extract was combined with sulfuric acid and acetic anhydride, and a color shift from violet to blue or green indicated the presence of steroids. Glycosides were indicated by the addition of sodium hydroxide and D to the extract, resulting in a yellow color [63]. Leucoanthocyanins were detected by mixing extract with isoamyl alcohol gave red color of the upper layer [64]. Lignin was identified by the formation of an olive green color after adding gallic acid to the extract [65]. Coumarins were detected by adding 10% sodium hydroxide to the extract, color turned yellow [66]. Fatty acids were detected by adding the extract to ether and then placing the mixture on a piece of filter paper and allowing the ether to evaporate and dry. A clear layer appeared on the filter paper, which was taken as evidence that fatty acids were present [67].

### 4.4 HPLC Analysis

Phytochemical analysis of the extract was performed using high‐performance liquid chromatography (HPLC) with an Agilent 1260 Infinity II instrument fitted with a diode array detector (DAD) for single detection at specific wavelengths (Agilent Technologies, Santa Clara, CA, USA). Separation was performed on a reverse‐phase C18 analytical column (Infinity Lab Poroshell, 100 × 4.6, 4 µm) by gradient elution. The mobile phase was 1.0% (v/v) formic acid in ultrapure water, denoted as solvent A) and acetonitrile, denoted as solvent B; it was maintained at a constant flow rate of 1.5 mL/min. A 5 µL sample of each sample was injected into the system. The DAD controlled the absorption at 254.4 and 326.4 nm for the sensitive detection of phenolic acids and flavonoids. This method enabled the precise separation and determination of important bioactive compounds present in the plant extract. The methodology was adapted from the standardized protocol described by [68] with few modifications.

### 4.5 GC‐MS Analysis

The chemical analysis and identification of bioactive compounds present in crude essential of *E. diffusum* was done by following the protocol of Nasir Shah et al., (2023) with few modifications [54]. The analytical method configured for the Agilent 5977B system was set to run for a total duration of 36 min. The oven temperature program began at 70°C, held steady for 3 min, then increased at a rate of 10°C per min until reaching 270°C, where it was held for an additional 13 min. A 1 µL sample was injected using an Auto Liquid Sampler (ALS) equipped with a 10 µL syringe, operating in split mode with a 20:1 split ratio. Helium was utilized as the carrier gas, maintained at a flow rate of 1 mL/min. The separation was performed on a DB‐1 capillary column (30 m length × 0.25 µm film thickness × 0.25 mm internal diameter), with a maximum operating temperature of 325°C. The transfer line to the Mass Selective Detector (MSD) was set to 280°C. Mass spectrometry was conducted in full scan mode, covering a mass‐to‐charge (m/z) range of 30 to 650 amu, with a 2‐min solvent delay. The ion source temperature was held at 230°C, while the quadrupole was maintained at 150°C. The electron multiplier voltage was applied using a gain factor of 1.0, and the instrument was operated in the standard scanning mode. Timed events and Trace Ion Detection (TID) were not employed in this method.

### 4.6 Antibacterial Activity

The antibacterial potential of the ethanolic extract of *E. diffusum* was determined against bacterial strains of *Staphylococcus epidermidis* (ATCC 12228), *Listeria monocytogenes* (ATCC 13932), *Staphylococcus aureus (ATTC 9144)*, *Pseudomonas aeruginosa* (ATCC 10145), *Bordetella bronchiseptica* (ATTC 4617), and *E. coli* (ATCC 10536) using the well diffusion method. The protocols of Khan et., (2023) and Assad et al., (2025) was adopted with some modifications for antibacterial assay [69, 70]. Mueller–Hinton (MH) agar was prepared by dissolving 6.0 g of MH powder in 120 mL of DW. The medium and sterile Petri dishes were autoclaved at 121°C for 15 min. After cooling to approximately 50°C, 25 mL of the sterilized agar was poured into each Petri dish and allowed to solidify for 20 min under aseptic conditions. Bacterial cultures were subcultured on nutrient agar and incubated at 35°C in a rotary shaker set at 200 rpm to ensure active growth. Inoculation onto MH agar plates was carried out using the streak plate technique. Sterile cork borers were employed to punch three equidistant wells in each plate, labeled as A, B, and C. Well A was loaded with 30 µg/mL of Erythromycin (positive control), while wells B and C were filled with 20 and 30 µg/mL of the plant extract, respectively. The plates were incubated at 37°C for 24 h. After incubation, the zones of inhibition around each well were measured in millimeters to assess antibacterial activity. All experimental steps were conducted within a laminar flow cabinet to maintain sterile conditions. Each test was carried out in triplicate, and the data are expressed as mean ± standard deviation.

The Minimum Inhibitory Concentration (MIC) and Minimum Bactericidal Concentration (MBC) of the ED extract were evaluated using the broth microdilution method [71]. Serial dilutions of the extract were made with sterile nutrient broth, 10 to 100 µg/mL. Each dilution was inoculated with adjusted bacterial suspensions (0.5 McFarland standard) and incubated at 37°C. for 24 h. MIC is the lowest concentration that prevents the growth of visible bacteria. To calculate the MBC, 100 µL from wells with no turbidity was transferred onto new agar plates and incubated under the same conditions. MBC was defined as the minimum concentration that led to 100% inhibition of bacterial colony formation.

### 4.7 Antioxidant Activity

#### 4.7.1 Ferric Reducing Antioxidant Power (FRAP) Assay

The FRAP assay was conducted according to the procedure described by Shobha et al. (2019) with slight changes [72]. Various doses of ED Plant extract solution (different concentrations of 100–500 µg/mL) were mixed with 2.5 mL of sodium phosphate buffer (PBS) (0.2 M, pH 6.6) 2.5 mL of 1% potassium ferricyanide and in incubated for 20 min at 50°C. The next step was the treatment with a 10% w/v trichloroacetic acid solution, followed by centrifugation at 3000 rpm for 10 min. A solution of 0.1% ferric chloride and 2.5 mL distilled water was added to the supernatant. A Shimadzu UV‐1800 spectrophotometer was used to record the absorbance at 700 nm. Equation (1) was used to estimate the FRAP antioxidant activity [73].

(1) $$X=\frac{y}{0.0027},\;R^{2}=0.9801\;\;\;\;\;\;\;\;\;\;\;\;\;\;$$

#### 4.7.2 Diphenyl‐1‐picrylhydrazyl (DPPH) Assay

The antioxidant capacity of the ED extract was assessed using the DPPH (2,2‐diphenyl‐1‐picrylhydrazyl) free radical scavenging test, following the methodology of Assad et al. (2024), with slight modifications. Various doses of the ED extract (100–500 µg/mL) were mixed with 2 mL of methanol and 1 mL of a 0.1 mM DPPH solution formulated in methanol. The mixtures were vortexed and incubated in the dark for 30 min. After incubation, the absorbance was measured at 517 nm using a Shimadzu UV‐1800 spectrophotometer. The antioxidant activity of the extract was quantified as the percentage of DPPH radical inhibition using Equation (2) [74].

(2) $$\begin{array}{cc} \%\;\mathrm{Scavenging}\;\mathrm{activity}\; & =\left(\mathrm{Ac}\;517\;\mathrm{of}\;\mathrm{control}\;-\;\mathrm{As}\;517\;\mathrm{of}\;\mathrm{sample}\right)\\ & /\;\mathrm{Ac}\;517\;\mathrm{of}\;\mathrm{control}\;\times \;100 \end{array}$$

#### 4.7.3 Total Flavonoid Content (TFC)

The total flavonoid content of the ED extract was quantified using a colorimetric technique with aluminum chloride (AlCl_3_), according to the methodology described by Bibi et al. (2026) with minor adjustments [75]. Different quantities of ED extract (100–500 µg/mL) were mixed with 0.75 mL methanol and diluted with distilled water to achieve a final volume of 2 mL. Subsequently, 300 µL of 5% sodium nitrite and 300 µL of 10% AlCl_3_ were incorporated into each sample. After a 10 min reaction at ambient temperature, 2 mL of 1 mol/L sodium hydroxide was added, and the liquid was diluted to a final volume of 5 mL using deionized water. After 40 min of incubation at room temperature, the absorbance was measured at 510 nm using a Shimadzu UV‐1800 spectrophotometer. Quercetin was used as the calibration standard, and the flavonoid concentration was quantified as milligrams of quercetin equivalents per gram of extract (mg QE/g). The relative flavonoid content was determined using Equation (3) [73].

(3) $$X=\frac{y}{0.0019},R^{2}=0.9659\;\;\;\;\;\;\;\;\;\;\;\;\;\;\;\;\;\;\;\;\;\;\;$$

### 4.8 Molecular Docking

Th*e* in silico molecular docking is one of the most appropriate methods for finding new ligands for proteins with known three‐dimensional structures and occupies a central place in the pharmaceutical field. Ligand–protein docking attempts to determine the primary binding mode of a ligand to a protein with a known three‐dimensional structure [76]. The molecular docking methodology used in this study involved several software and tool.

#### 4.8.1 Retrieval of Targeted Protein

First, the crystal structures of the antimicrobial and antioxidant target proteins were retrieved from the RCSB protein data bank (https://www.rcsb.org) [77]. The PDB IDs of these proteins were 1R4U and 1AJ6, respectively. In this study, the crystal structures of two proteins (PDB IDs: 1AJ6 and 1R4U) were utilized for molecular docking as a result of being readily available, biofunctionally relevant and diverse targets. Their choice is directed by documented biological purposes, structural properties, health‐associated parts or historical predictions, and the capability to reveal possible new connections. In addition, these proteins have sites that bind to metabolic enzymes, providing insights into general cellular processes. Docking targets were selected based on the availability of the software tools. The BIOVIA Discovery Studio Visualizer and Auto Dock tools were used to prepare the proteins for docking [78, 79, 80, 81].

#### 4.8.2 Identification of Ligands

All GC‐MS‐identified ligand 3D models were downloaded in SDF format, and the PubChem ID, molecular weight, molecular formula, and conical smiles were obtained from the PubChem database (https://pubchem.ncbi.nlm.nih.gov/) [82] (Table 7). The ligands were carefully designed and illustrated in ChemDraw, followed by energy minimization using Avogadro software. The ligands were subsequently transformed into pdbqt files using AutoDock. A grid box was constructed in AutoDock, and its center was fine‐tuned to encompass the active pocket. AutoDock Vina was used for the docking procedure [83]. The ligands were docked into the active site of the protein, and the ligand–protein interactions that were produced as a consequence were examined using the Discovery Studio Visualizer available via BIOVIA [84].

**TABLE 7:** Physiochemical properties, druglikeness, and pharmacokinetic properties of selected novel ligands, 7,9‐di‐*tert*‐butyl‐1‐oxaspiro (4, 5) deca‐6,9‐diene‐2,8‐dione.

Physiochemical properties		Drug likeness		Pharmacokinetics	
Fraction Csp3	0.66	Lipinski	Yes	GI absorption	High
Num.rotatable bonds	2	Ghose	Yes	P‐gp substrate	Yes
Num.H‐bonds acceptors	3	Veber	Yes	BBB permeant	No
Num.H‐bonds donors	0	Egan	Yes	CYP1A2 inhibitor	No
Molar refractivity	79.66	Muegge	Yes	CYP2C19 inhibitor	Yes
TPSA	43.37 Å^2^	Bioavailability score	0.55	Log k_p_(skin permeability)	−5.28 cm/s

The 2D structures were drawn using ChemDraw, and the energy minimization of the designed and prepared compounds was performed using Avogadro. The SDF 3D models were retrieved from PubChem (https://pubchem.ncbi.nlm.nih.gov/). Using AutoDock, the ligands were converted to pdbqt files. A grid box was created in AutoDock, and the center of the grid box was treated to include the active site. AutoDock Vina was used for docking. Ligand–protein interactions were analyzed by docking the ligands into the active site of the protein using BIOVIA Discovery Studio Visualizer [85].

### 4.9 ADMET Analysis

Preclinical testing of the drug candidate was conducted using the SwissADME tool (http://www.swissadme.ch/) [86]. using the Pro Tox II server (https://tox‐new.charite.de/protox_II/index.php?site = home), the ligand's toxicity was examined [87]. For the docking study of the chosen best‐screen active compounds, Autodock Vina, an open‐source program, was utilized [50, 55]. (Table 8).

**TABLE 8:** GC‐MS identified phytochemicals with their PDB ID, MW, MF, and conical smiles.

Phytochemicals	CID	MW	MF	Smiles
Oleic acid	445639	282.5 g/mol	C18H34O	CCCCCCCC/C═C∖CCCCCCCC(═O)O
n‐Hexadecanoicacid	985	256.42 g/mol	C16H32O2	CCCCCCCCCCCCCCCC(═O)O
Methyl elaidate	5280590	296.5 g/mol	C19H36O2	CCCCCCCC/C ═C/CCCCCCCC(═O)OC
Hexadecanoic acid methyl ester	8181	270.5 g/mol	C17H34O2	CCCCCCCCCCCCCCCC(═O)OC
Bis(2‐ethylhexyl) terephthalate	22932	390.6 g/mol	C24H38O4	CCCCC(CC)COC(═O)C1 ═CC = C(C= C1)C(═O)OCC(CC)CCCC
Bis(2‐ethylhexyl) phthalate	8343	390.6 g/mol	C24H38O4	CCCCC(CC)COC(═O)C1 ═CC ═CC═ C1C(═O)OCC(CC)CCCC
10,13‐Octadecadienoic acid, methyl ester	5365678	294.5 g/mol	C19H34O2	CCCC/C ═C/C/C═C/CCCCCCCCC(═ O)OC
9‐octadecenoic acid methyl ester	8202	296.5 g/mol	C19H36O2	CCCCCCCC/C═C∖CCCCCCCC(═O)OC
7,9‐Di‐*tert*‐butyl‐1‐oxaspiro(4,5)deca‐6,9‐diene‐2,8‐dione	545303	276.4 g/mol	C17H24O3	CC(C)(C)C1═CC2(CCC(═O)O2)C═C(C1 ═O)C(C)(C)C
2 4‐di‐*tert*‐butylphenol	7311	206.32 g/mol	C14H22O	CC(C)(C)C1═CC(═C(C ═C1)O)C(C)(C)C

### 4.10 Statistical Analysis

All experiments were performed in triplicate (*n* = 3), and data are presented as the mean ± standard deviation (SD). To evaluate the significance of the findings, a one‐way analysis of variance (ANOVA) was conducted, followed by Tukey's post‐hoc test for multiple comparisons. This was specifically employed to determine significant differences (*p* < 0.05) in the zones of inhibition between the *E. diffusum* extract concentrations and the positive control (Erythromycin). For the antioxidant assays (DPPH, FRAP, and TFC), a two‐way ANOVA was utilized to assess the influence of two independent variables—concentration and treatment type—on the observed antioxidant capacity. All statistical calculations and graph generation were performed using GraphPad Prism (version 8.0, GraphPad Software, USA), Statstix (version 8.1), and OriginPro (version 2021). Differences were considered statistically significant at *p* < 0.05.

## Author Contributions

Conceptualization: N.A., M.N.U.H.; Data curation: M.F.U.R., Y.A.; Formal analysis: Y.A., H.G., M.B.L.; Investigation: M.F.U.R., H.G.; Methodology: M.N.K., M.B.L., Z.N.; Project administration: A.K., M.A., M.N.K.; Resources: Z.N., M.A.; Software: A.K., E.G.G.; Validation: N.A.; Writing – original draft: N.A., M.N.U.H.; Writing – review and editing: S.E., A.K., E.G.G. All authors have read and agreed to the published version of the manuscript.

## Funding

This research was supported by the Higher Education Commission (HEC), Pakistan, under the Indigenous Scholarship Program (Scholarship ID: 520‐161352‐2PS6‐111; Reference No.: 50093302).

## Consent to Publish

All authors have read and approved the final manuscript and consented to its publication.

## Conflicts of Interest

The authors declare no conflicts of interest.

## Supporting information



The supporting information includes Supporting File 1: Complete results in PowerPoint format; Supporting File 2: sequence file (Chromatogram ab1); Supporting File 3: sequence file (Sequence Quality Report and Blast); Supporting File 4: sequence file (Sequence Signal Peaks); Supporting File 5: HPLC results; Supporting File 6: GC–MS chromatogram.
**Supporting File 1**: cbdv71870‐sup‐0001‐Fig. S1‐S2.docx

**Supporting File 2**: cbdv71870‐sup‐0002‐Supporting File 2.ab1

**Supporting File 3**: cbdv71870‐sup‐0003‐Supporting File 3.csv

**Supporting File 4**: cbdv71870‐sup‐0004‐Supporting File 4.pdf

**Supporting File 5**: cbdv71870‐sup‐0005‐Supporting File 5.pdf

**Supporting File 6**: cbdv71870‐sup‐0006‐Supporting File 6.pdf

**Supporting File 7**: cbdv71870‐sup‐0007‐Data.zip

## Data Availability

All data generated or analyzed during this study are included in this article.
