# Supplementary material for: Integrated Phylogenetic, Phytochemical, and In Silico Evaluation of Equisetum diffusum D. Don. Ethanolic Extract for Bioactivity
Source: Chem Biodivers. 2026 Oct 26;23(10):e02873. doi: 10.1002/cbdv.202502873 (PMC13698640; doi:10.1002/cbdv.202502873)
Supplement: Supplementary file 1 [file CBDV-23-e02873-s002.pdf]

Area % Report

Data File: D:\HPLC DATA\FP\EtOH 326 ED 955.dat
Method: C:\EZChrom Elite\Enterprise\Projects\Nutrabiotics\Method\Plant Reserch Method..met
Acquired: 8/1/2024 1:51:55 AM
Printed: 8/1/2024 2:26:51 AM

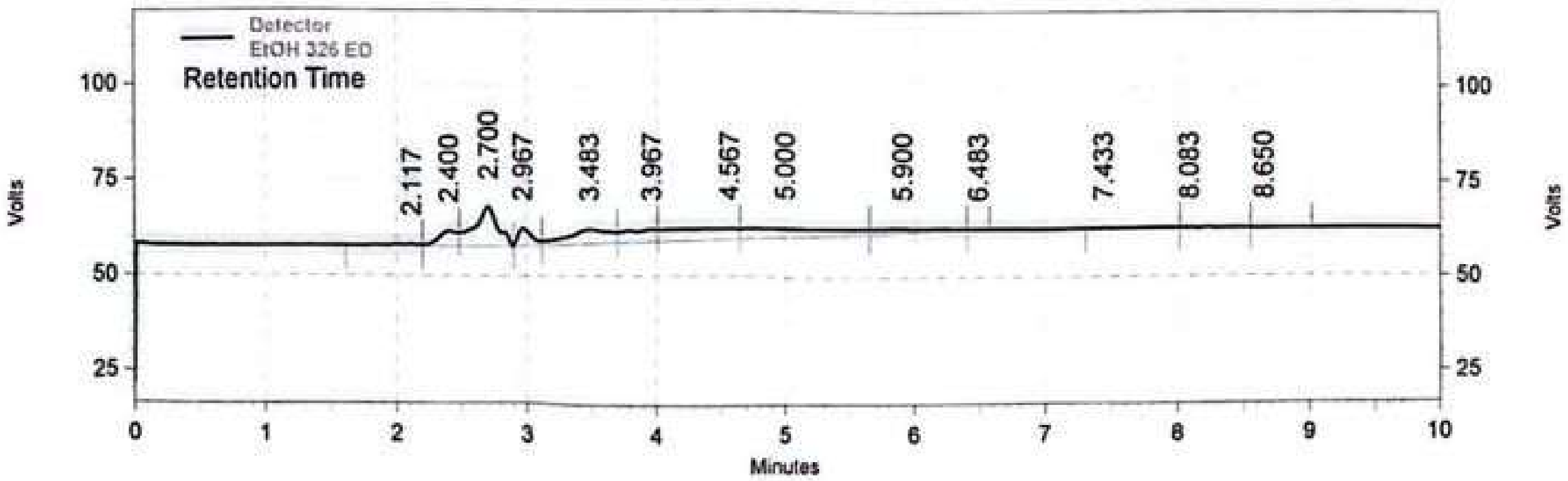

Detector Results

Retention Time	Area	Area %	Height	Height %
2.117	9152	0.74	590	0.92
2.400	79459	6.41	7984	12.42
2.700	258650	20.88	20399	31.74
2.967	65787	5.31	8875	13.81
3.483	175644	14.18	7450	11.59
3.967	116375	9.39	6617	10.30
4.567	222260	17.94	5493	8.55
5.000	234194	18.91	4344	6.76
5.900	71557	5.78	2093	3.26
6.483	2468	0.20	262	0.41
7.433	2107	0.17	87	0.14
8.083	686	0.06	44	0.07
8.650	430	0.03	28	0.04
Totals	1238769	100.00	64266	100.00

Scanned with CamScanner

Area % Report

Data File: D:\HPLC DATA\FP\Nasir EthOH ED952.dat
Method: C:\EZChrom Elite\Enterprise\Projects\Nutrabiotics\Method\Plant Reserch Method..met
Acquired: 8/1/2024 12:40:10 AM
Printed: 8/1/2024 12:54:55 AM

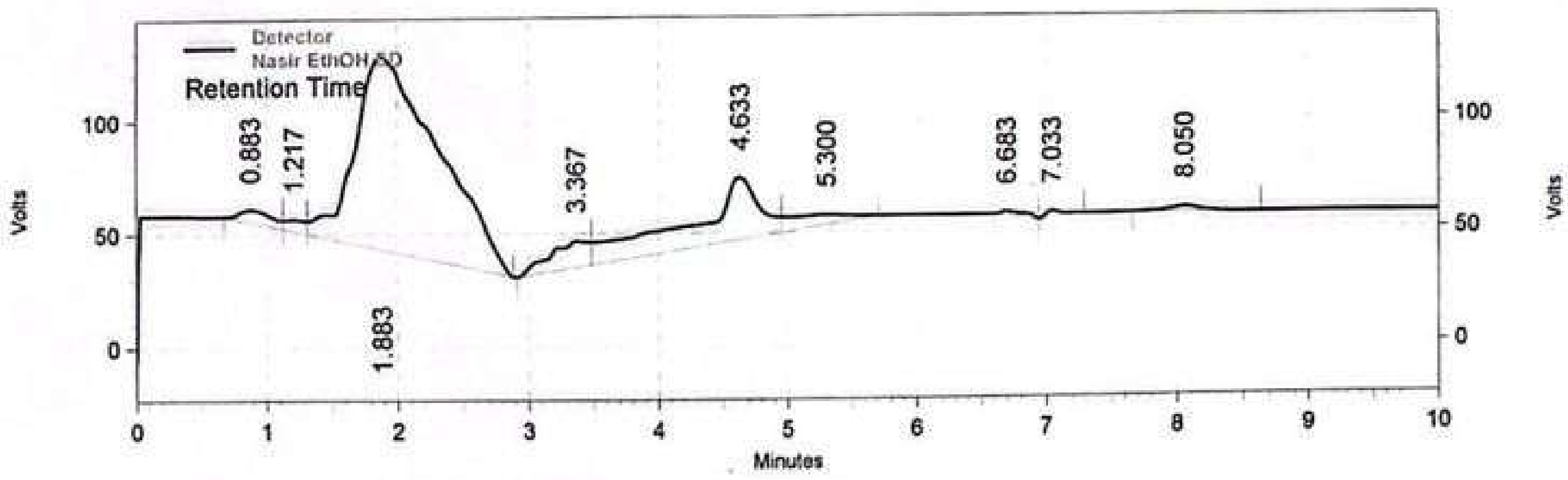

Detector Results

Retention Time	Area	Area %	Height	Height %
0.883	207345	1.89	11125	3.81
1.217	110844	1.01	10278	3.52
1.883	7596275	69.36	170464	58.39
3.367	489746	4.47	23398	8.01
4.633	2041755	18.64	54167	18.55
5.300	321200	2.93	8283	2.84
6.683	56751	0.52	3807	1.30
7.033	50361	0.46	5758	1.97
8.050	78193	0.71	4664	1.60
Totals	10952470	100.00	291944	100.00

CS Scanned with CamScanner
