# Supplementary material for: Integrated Phylogenetic, Phytochemical, and In Silico Evaluation of Equisetum diffusum D. Don. Ethanolic Extract for Bioactivity
Source: Chem Biodivers. 2026 Oct 26;23(10):e02873. doi: 10.1002/cbdv.202502873 (PMC13698640; doi:10.1002/cbdv.202502873)
Supplement: Supplementary file 2 [file CBDV-23-e02873-s003.pdf]

Library Search Report

```
Data Path : D:\CRL CHEMICALS NEW\9488 NASIR ASAD UNI OF SARGODHA\
Data File : 9488 NASIR ASAD UNI OF SARGODHA ETHANOL.D
Acq On    : 01 Feb 2024  14:35
Operator  : CRL
Sample    : ETHANOL
Misc      :
ALS Vial  : 4   Sample Multiplier: 1

Search Libraries:   C:\MassHunter\LIBRARY\NIST20.L     Minimum Quality:   0

Unknown Spectrum:   Apex
Integration Events: ChemStation Integrator - autoint1.e
```

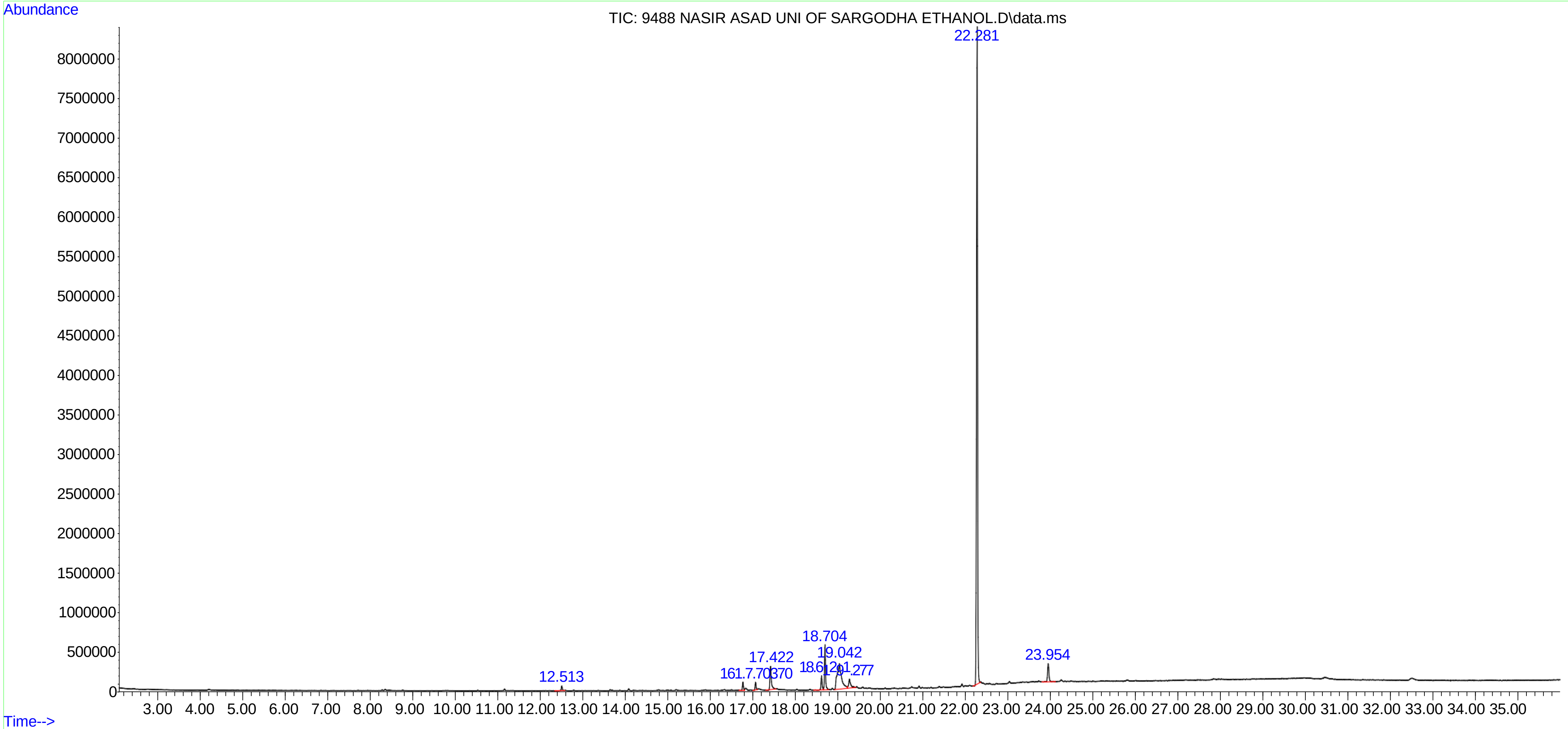

Unknown Spectrum based on Apex

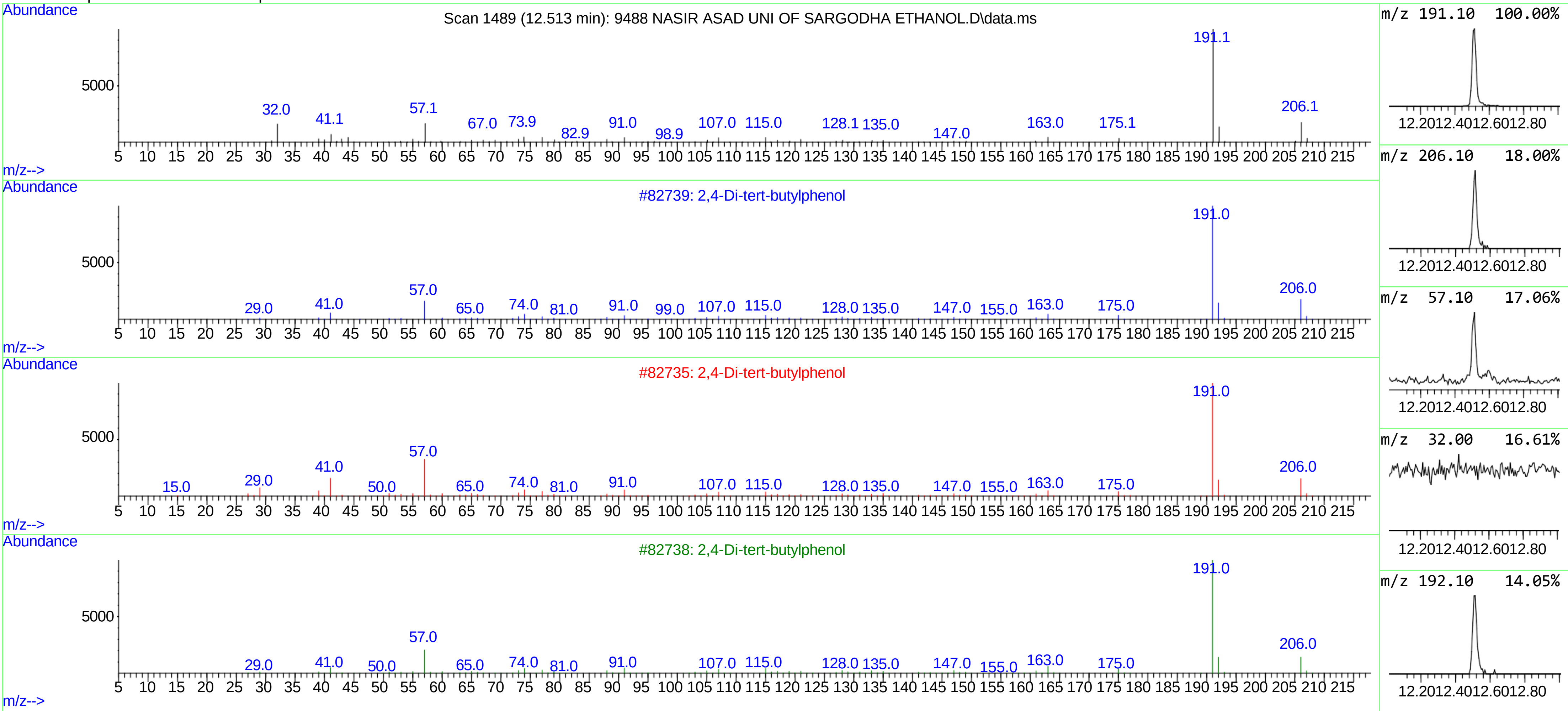

Data File: D:\CRL CHEMICALS NEW\9488 NASIR ASAD UNI OF SARGODHA\9488 NASIR ASAD UNI OF SARGODHA ETHANOL.D
Sample : ETHANOL

Peak Number: 1 at 12.513 min Area: 1090462 Area % 0.54

The 3 best hits from each library.	Ref\#	CAS\#	Qual
C:\MassHunter\LIBRARY\NIST20.L			
1 2,4-Di-tert-butylphenol	82739	000096-76-4	96
2 2,4-Di-tert-butylphenol	82735	000096-76-4	95
3 2,4-Di-tert-butylphenol	82738	000096-76-4	94

Unknown Spectrum based on Apex

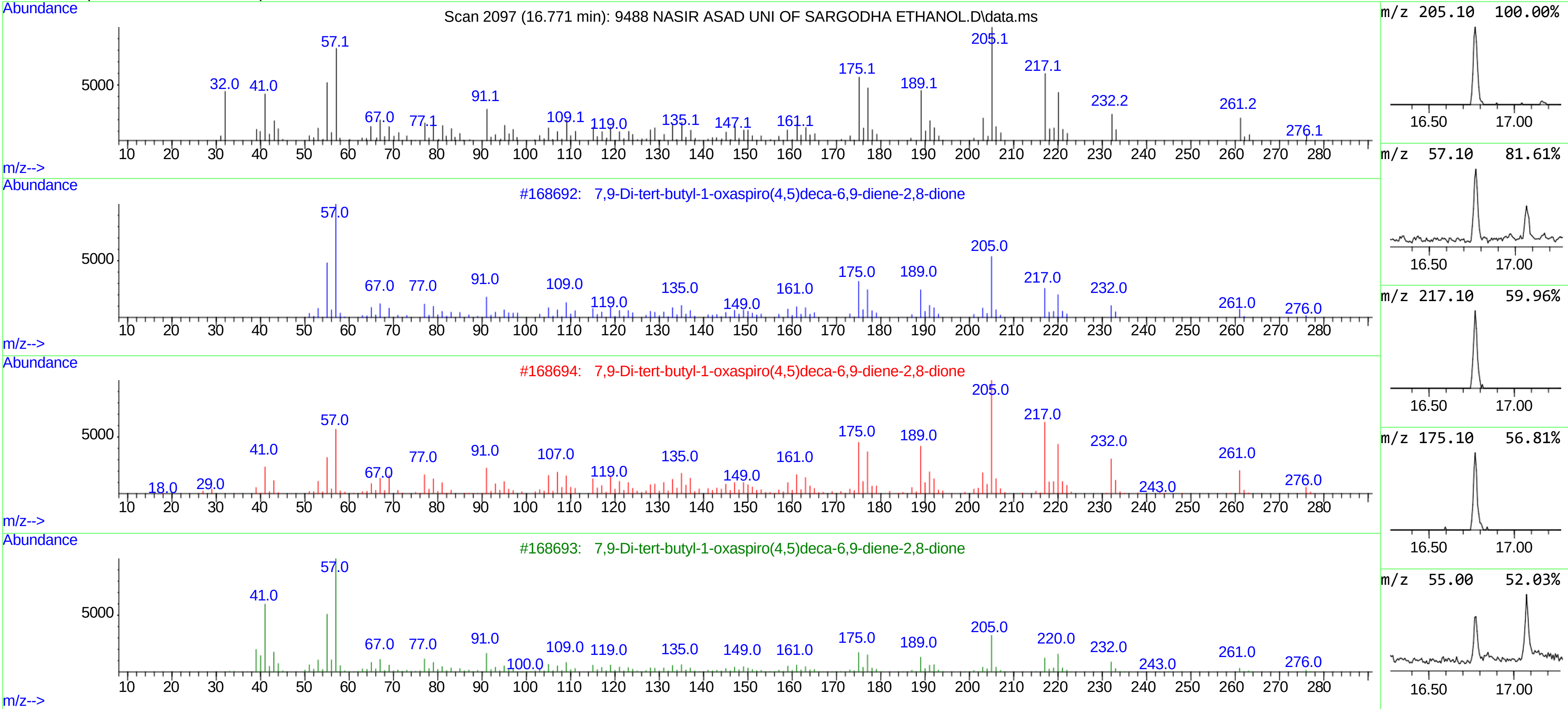

Data File: D:\CRL CHEMICALS NEW\9488 NASIR ASAD UNI OF SARGODHA\9488 NASIR ASAD UNI OF SARGODHA ETHANOL.D
Sample : ETHANOL

Peak Number: 2 at 16.773 min Area: 1722225 Area % 0.85

The 3 best hits from each library.	Ref\#	CAS\#	Qual
C:\MassHunter\LIBRARY\NIST20.L			
1 7,9-Di-tert-butyl-1-oxaspiro(4,5...	168692	082304-66-3	99
2 7,9-Di-tert-butyl-1-oxaspiro(4,5...	168694	082304-66-3	95
3 7,9-Di-tert-butyl-1-oxaspiro(4,5...	168693	082304-66-3	94

Unknown Spectrum based on Apex

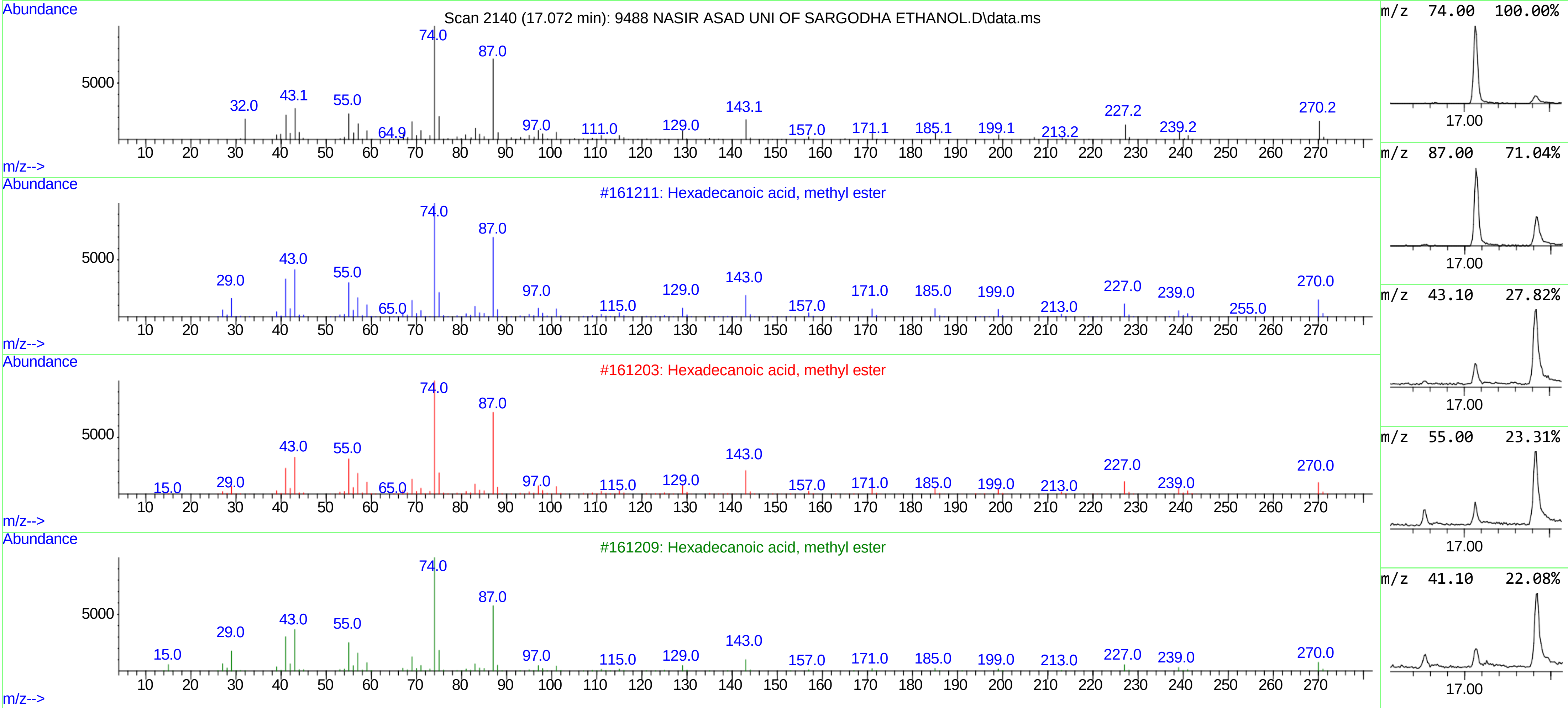

Data File: D:\CRL CHEMICALS NEW\9488 NASIR ASAD UNI OF SARGODHA\9488 NASIR ASAD UNI OF SARGODHA ETHANOL.D
Sample : ETHANOL

Peak Number: 3 at 17.070 min Area: 1546457 Area % 0.76

The 3 best hits from each library.	Ref#	CAS#	Qual
C:\MassHunter\LIBRARY\NIST20.L			
1 Hexadecanoic acid, methyl ester	161211	000112-39-0	99
2 Hexadecanoic acid, methyl ester	161203	000112-39-0	99
3 Hexadecanoic acid, methyl ester	161209	000112-39-0	97

Unknown Spectrum based on Apex

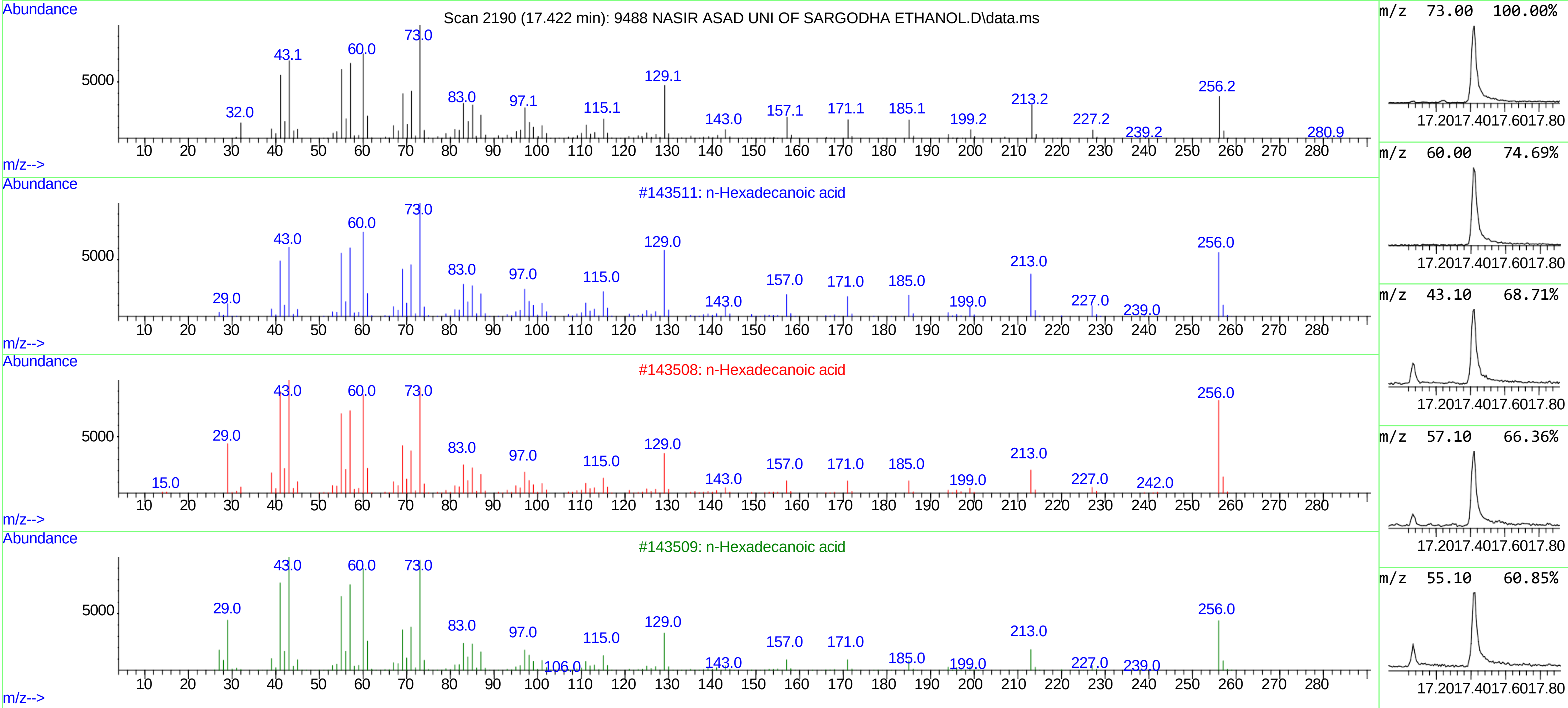

Data File: D:\CRL CHEMICALS NEW\9488 NASIR ASAD UNI OF SARGODHA\9488 NASIR ASAD UNI OF SARGODHA ETHANOL.D
Sample : ETHANOL

Peak Number: 4 at 17.422 min Area: 6745997 Area % 3.34

The 3 best hits from each library.	Ref\#	CAS\#	Qual
C:\MassHunter\LIBRARY\NIST20.L			
1 n-Hexadecanoic acid	143511	000057-10-3	99
2 n-Hexadecanoic acid	143508	000057-10-3	99
3 n-Hexadecanoic acid	143509	000057-10-3	99

Unknown Spectrum based on Apex

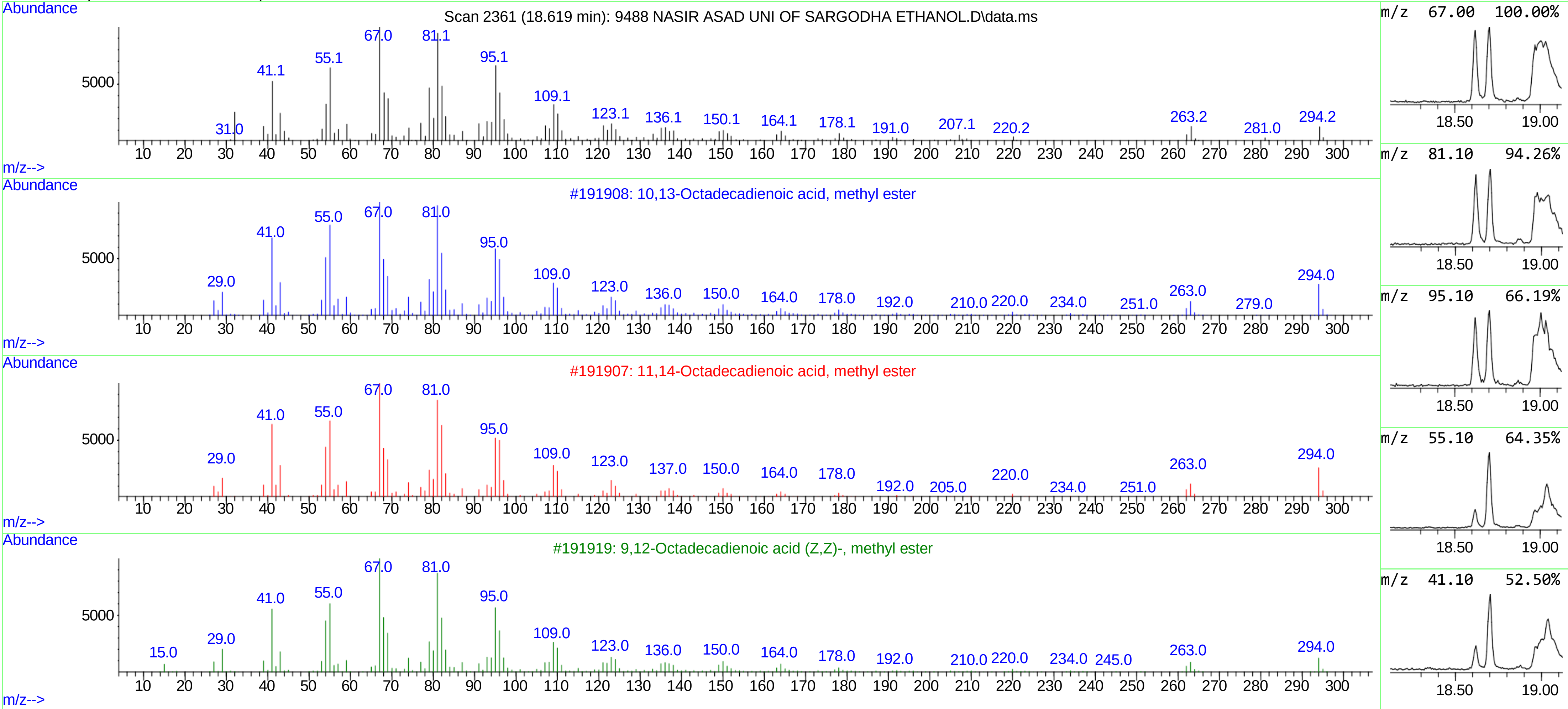


```
Data File: D:\CRL CHEMICALS NEW\9488 NASIR ASAD UNI OF SARGODHA\9488 NASIR ASAD UNI OF SARGODHA ETHANOL.D
Sample    : ETHANOL

Peak Number:  5      at     18.621 min  Area:    3154064  Area % 1.56

 The 3 best hits from each library.          Ref\#     CAS\#       Qual
-----------------------------------------------------------------------------------------------------------------
    C:\MassHunter\LIBRARY\NIST20.L
     1 10,13-Octadecadienoic acid, meth...      191908 056554-62-2    99
     2 11,14-Octadecadienoic acid, meth...      191907 056554-61-1    99
     3 9,12-Octadecadienoic acid (Z,Z)-...      191919 000112-63-0    98
```

Unknown Spectrum based on Apex

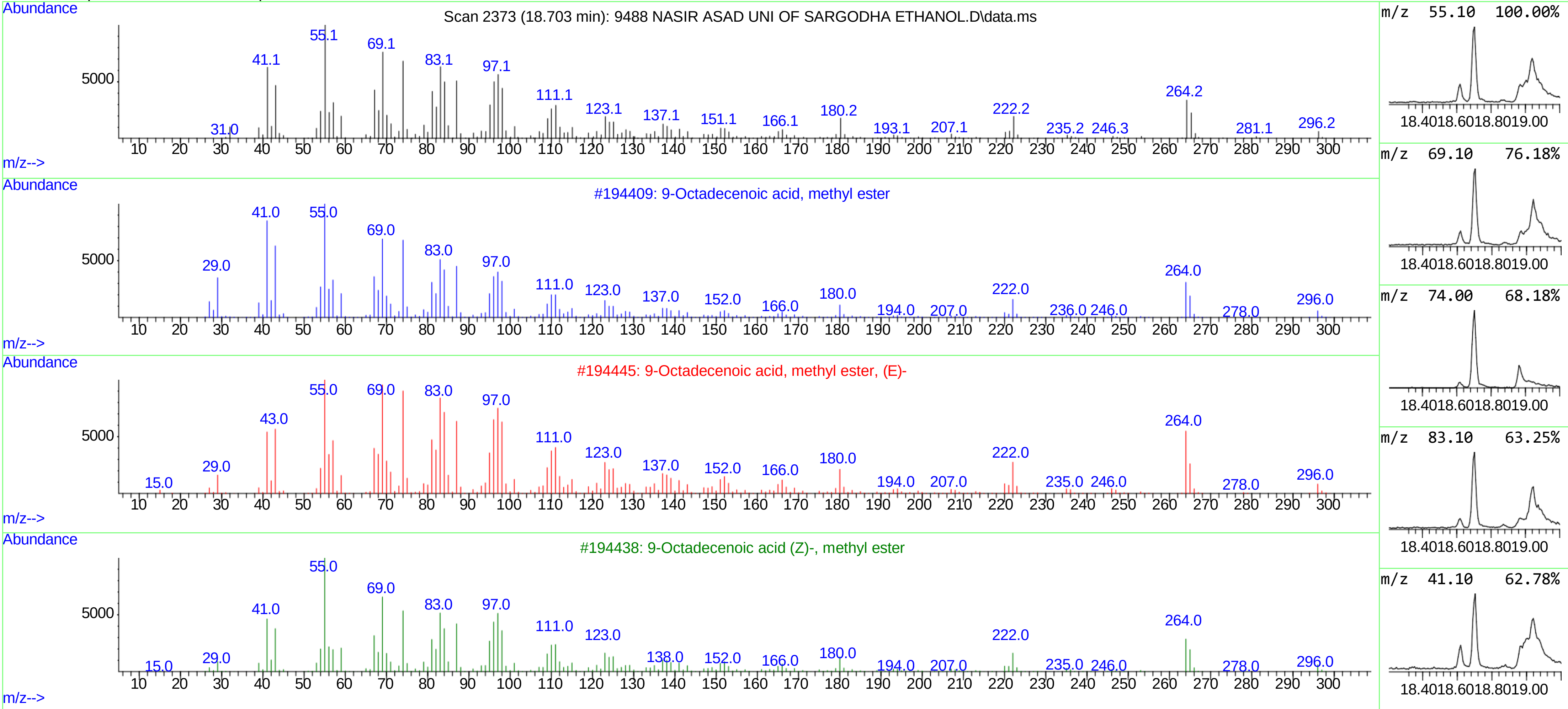

Data File: D:\CRL CHEMICALS NEW\9488 NASIR ASAD UNI OF SARGODHA\9488 NASIR ASAD UNI OF SARGODHA ETHANOL.D
Sample : ETHANOL

Peak Number: 6 at 18.704 min Area: 10057523 Area % 4.97

The 3 best hits from each library.	Ref\#	CAS\#	Qual
C:\MassHunter\LIBRARY\NIST20.L			
1 9-Octadecenoic acid, methyl ester	194409	002462-84-2	99
2 9-Octadecenoic acid, methyl este...	194445	001937-62-8	99
3 9-Octadecenoic acid (Z)-, methyl...	194438	000112-62-9	99

Unknown Spectrum based on Apex

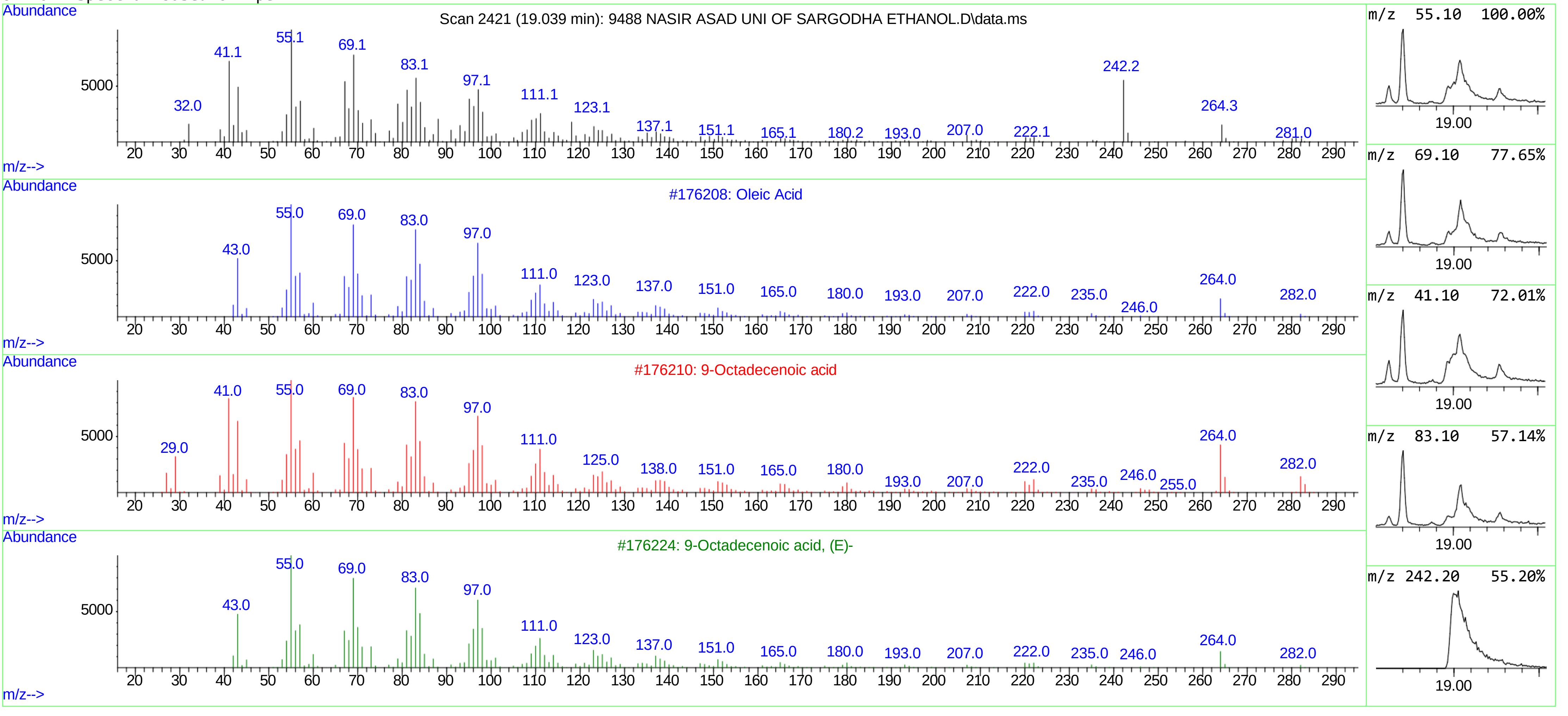

Data File: D:\CRL CHEMICALS NEW\9488 NASIR ASAD UNI OF SARGODHA\9488 NASIR ASAD UNI OF SARGODHA ETHANOL.D
Sample : ETHANOL

Peak Number: 7 at 19.042 min Area: 22241586 Area % 11.00

The 3 best hits from each library.	Ref\#	CAS\#	Qual
C:\MassHunter\LIBRARY\NIST20.L			
1 Oleic Acid	176208	000112-80-1	99
2 9-Octadecenoic acid	176210	002027-47-6	99
3 9-Octadecenoic acid, (E)-	176224	000112-79-8	98

Unknown Spectrum based on Apex

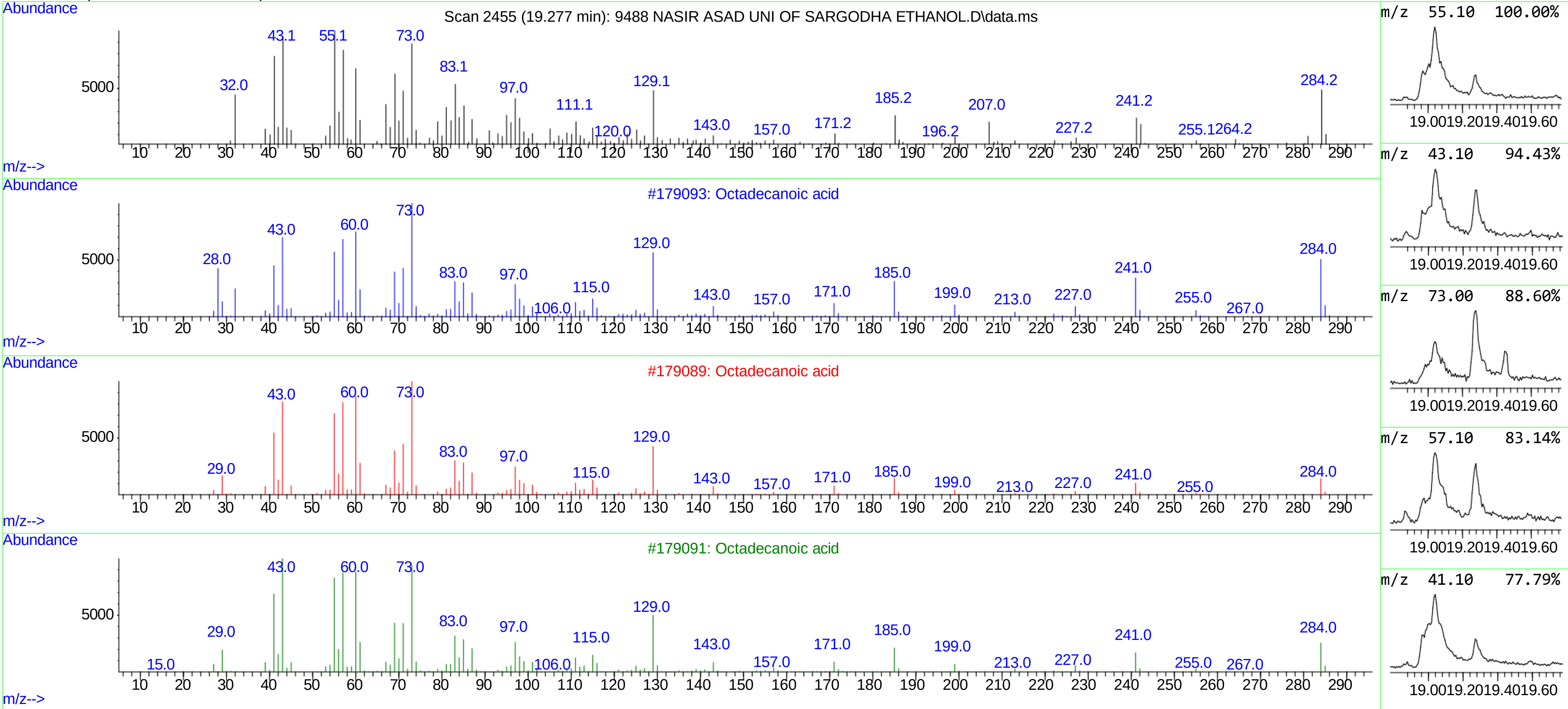

Data File: D:\CRL CHEMICALS NEW\9488 NASIR ASAD UNI OF SARGODHA\9488 NASIR ASAD UNI OF SARGODHA ETHANOL.D
Sample : ETHANOL

Peak Number: 8 at 19.277 min Area: 3972153 Area % 1.96

The 3 best hits from each library.	Ref\#	CAS\#	Qual
C:\MassHunter\LIBRARY\NIST20.L			
1 Octadecanoic acid	179093	000057-11-4	98
2 Octadecanoic acid	179089	000057-11-4	97
3 Octadecanoic acid	179091	000057-11-4	95

Unknown Spectrum based on Apex

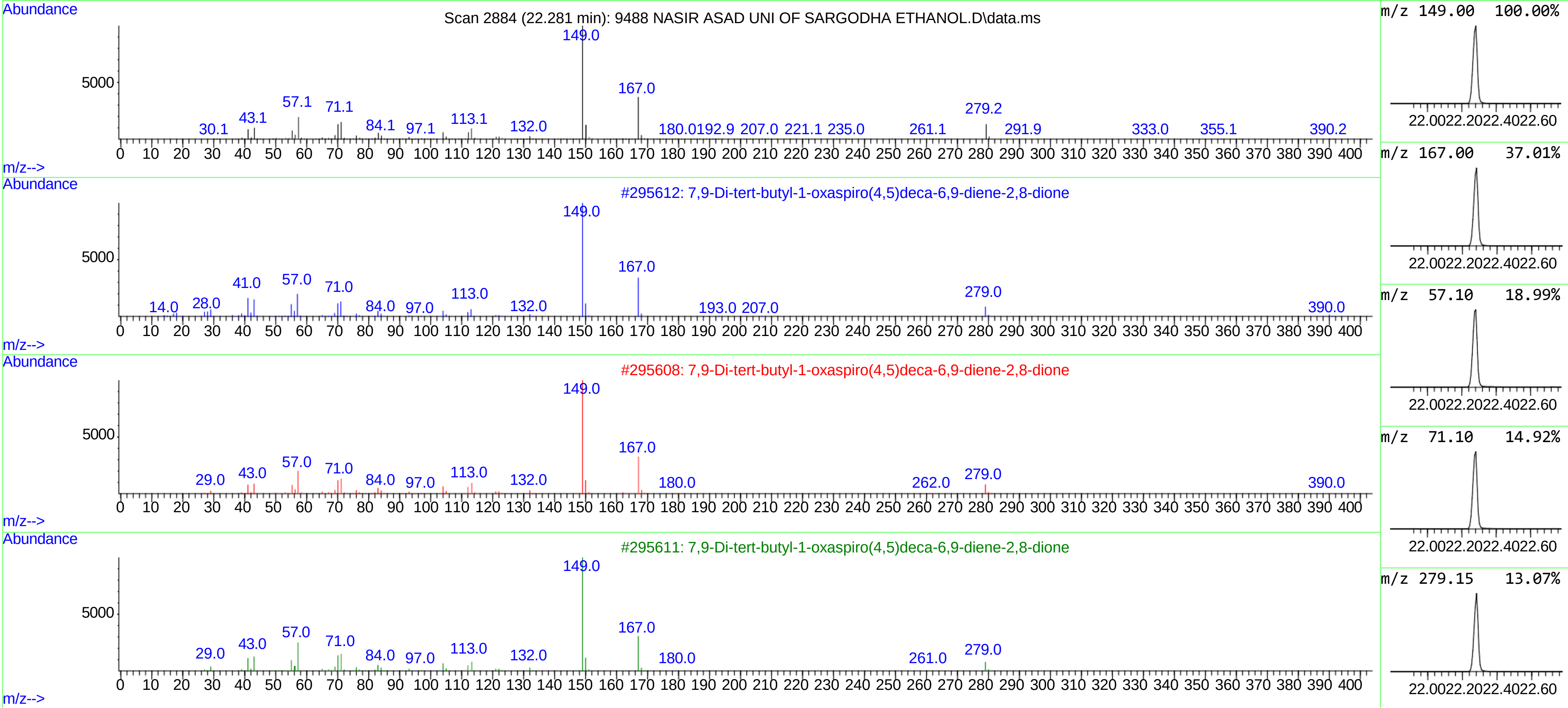

Data File: D:\CRL CHEMICALS NEW\9488 NASIR ASAD UNI OF SARGODHA\9488 NASIR ASAD UNI OF SARGODHA ETHANOL.D
Sample : ETHANOL

Peak Number: 9 at 22.281 min Area: 146241457 Area % 72.32

The 3 best hits from each library.	Ref\#	CAS\#	Qual
C:\MassHunter\LIBRARY\NIST20.L			
1 7,9-Di-tert butyl-1-oxaspiro...	545303	82304-66-3	91
2 7,9-Di-tert butyl-1-oxaspiro...	545303	82304-66-3	91
3 7,9-Di-tert butyl-1-oxaspiro...	545303	82304-66-3	91

Library Search Report - ChemStation Integrator

Unknown Spectrum based on Apex

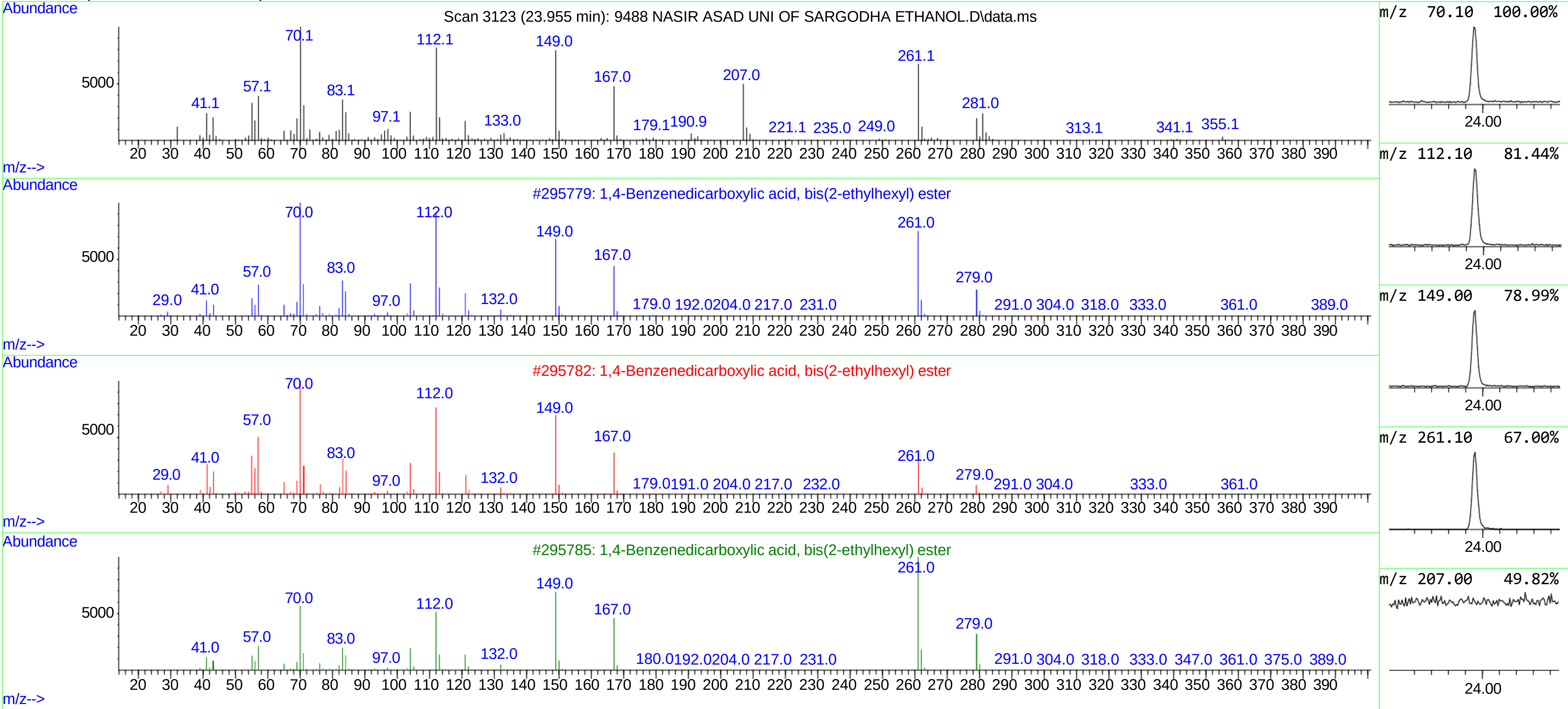

Data File: D:\CRL CHEMICALS NEW\9488 NASIR ASAD UNI OF SARGODHA\9488 NASIR ASAD UNI OF SARGODHA ETHANOL.D
Sample : ETHANOL

Peak Number: 10 at 23.954 min Area: 5434035 Area % 2.69

The 3 best hits from each library.	Ref\#	CAS\#	Qual
C:\MassHunter\LIBRARY\NIST20.L			
1 1,4-Benzenedicarboxylic acid, bi...	295779	006422-86-2	93
2 1,4-Benzenedicarboxylic acid, bi...	295782	006422-86-2	81
3 1,4-Benzenedicarboxylic acid, bi...	295785	006422-86-2	81
