# Supplementary material for: Integrated Phylogenetic, Phytochemical, and In Silico Evaluation of Equisetum diffusum D. Don. Ethanolic Extract for Bioactivity
Source: Chem Biodivers. 2026 Oct 26;23(10):e02873. doi: 10.1002/cbdv.202502873 (PMC13698640; doi:10.1002/cbdv.202502873)
Supplement: Supplementary file 3 [file CBDV-23-e02873-s007.pdf]

## Area % Report

Data File: D:\HPLC DATA\FP\EtOH 326 ED 955.dat
Method: C:\EZChrom Elite\Enterprise\Projects\Nutrabiotics\Method\Plant Reserch Method..met
Acquired: 8/1/2024 1:51:55 AM
Printed: 8/1/2024 2:26:51 AM

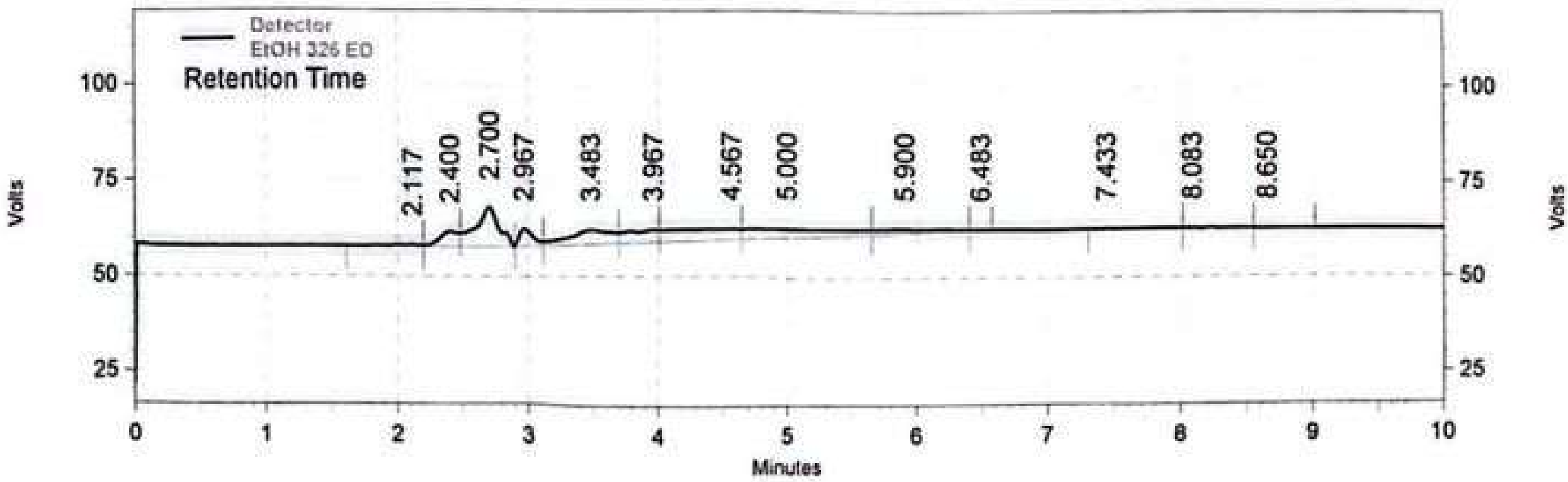


### Detector Results

| Retention Time | Area | Area % | Height | Height % |
|---|---|---|---|---|
| 2.117 | 9152 | 0.74 | 590 | 0.92 |
| 2.400 | 79459 | 6.41 | 7984 | 12.42 |
| 2.700 | 258650 | 20.88 | 20399 | 31.74 |
| 2.967 | 65787 | 5.31 | 8875 | 13.81 |
| 3.483 | 175644 | 14.18 | 7450 | 11.59 |
| 3.967 | 116375 | 9.39 | 6617 | 10.30 |
| 4.567 | 222260 | 17.94 | 5493 | 8.55 |
| 5.000 | 234194 | 18.91 | 4344 | 6.76 |
| 5.900 | 71557 | 5.78 | 2093 | 3.26 |
| 6.483 | 2468 | 0.20 | 262 | 0.41 |
| 7.433 | 2107 | 0.17 | 87 | 0.14 |
| 8.083 | 686 | 0.06 | 44 | 0.07 |
| 8.650 | 430 | 0.03 | 28 | 0.04 |
| Totals | 1238769 | 100.00 | 64266 | 100.00 |

Scanned with CamScanner

## Area % Report

Data File: D:\HPLC DATA\FP\Nasir EthOH ED952.dat
Method: C:\EZChrom Elite\Enterprise\Projects\Nutrabiotics\Method\Plant Reserch Method..met
Acquired: 8/1/2024 12:40:10 AM
Printed: 8/1/2024 12:54:55 AM

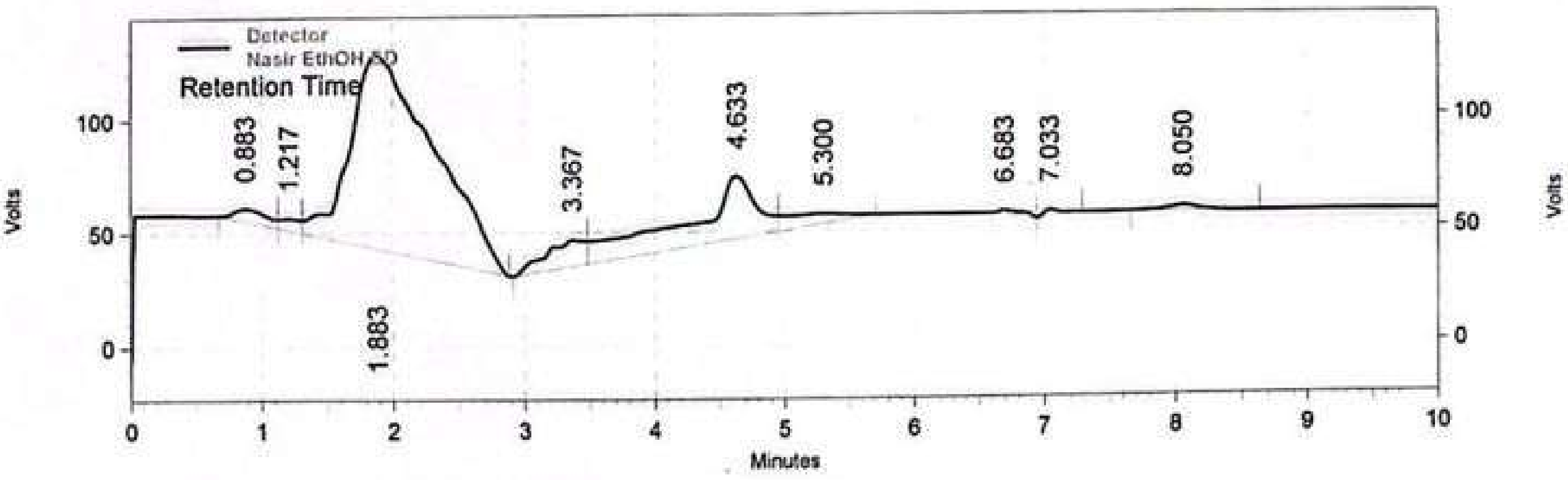


### Detector Results

| Retention Time | Area | Area % | Height | Height % |
|---|---|---|---|---|
| 0.883 | 207345 | 1.89 | 11125 | 3.81 |
| 1.217 | 110844 | 1.01 | 10278 | 3.52 |
| 1.883 | 7596275 | 69.36 | 170464 | 58.39 |
| 3.367 | 489746 | 4.47 | 23398 | 8.01 |
| 4.633 | 2041755 | 18.64 | 54167 | 18.55 |
| 5.300 | 321200 | 2.93 | 8283 | 2.84 |
| 6.683 | 56751 | 0.52 | 3807 | 1.30 |
| 7.033 | 50361 | 0.46 | 5758 | 1.97 |
| 8.050 | 78193 | 0.71 | 4664 | 1.60 |
| Totals | 10952470 | 100.00 | 291944 | 100.00 |

Scanned with CamScanner
