# Supplementary material for: Integrated Phylogenetic, Phytochemical, and In Silico Evaluation of Equisetum diffusum D. Don. Ethanolic Extract for Bioactivity
Source: Chem Biodivers. 2026 Oct 26;23(10):e02873. doi: 10.1002/cbdv.202502873 (PMC13698640; doi:10.1002/cbdv.202502873)

BIO TRACE
BioEdit version 7.7.1 (5/10/2021)

Model 1
(ý”
6258050-03 625800Asad Bashir-pb3.6258003-03 6258005-00

File: Chromatogram.ab1
BIF
Lane 0

Signal G:1506 A:1205 T:1009 C:1268
(ý”
?? no 'MTXF' field
Points 1689 to 15960

1/1/9999
Spacing: 14.1021747589111

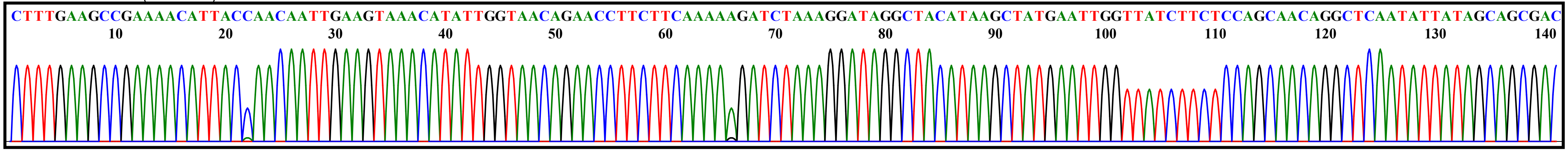

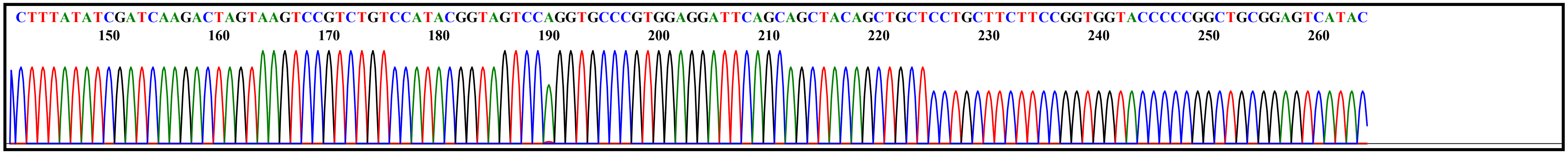

Supplement: Supplementary file 4 [file CBDV-23-e02873-s001.zip › Sequence Signal Peaks.pdf]
