# Supplementary material for: Integrated Phylogenetic, Phytochemical, and In Silico Evaluation of Equisetum diffusum D. Don. Ethanolic Extract for Bioactivity
Source: Chem Biodivers. 2026 Oct 26;23(10):e02873. doi: 10.1002/cbdv.202502873 (PMC13698640; doi:10.1002/cbdv.202502873)
Supplement: Supplementary file 5 [file CBDV-23-e02873-s006.docx]

Figure 1S: *E. diffusum* plant

Figure 2S: Agarose gel electrophoresis.
